# Biomolecular Microneedle Initiates Fe_3_O_4_/MXene Heterojunction‐Mediated Nanozyme‐Like Reactions and Bacterial Ferroptosis to Repair Diabetic Wounds

**DOI:** 10.1002/advs.202417314

**Published:** 2025-01-23

**Authors:** Wenjie You, Zichao Cai, Feng Xiao, Jiaxin Zhao, Guanyi Wang, Wang Wang, Zesheng Chen, Weikang Hu, Yun Chen, Zijian Wang

**Affiliations:** ^1^ Department of Urology, Institute of Urology Cancer Precision Diagnosis and Treatment and Translational Medicine Hubei Engineering Research Center Zhongnan Hospital of Wuhan University Wuhan 430071 China; ^2^ Department of Biomedical Engineering Hubei Province Key Laboratory of Allergy and Immune Related Disease TaiKang Medical School (School of Basic Medical Sciences) Wuhan University Wuhan 430071 China; ^3^ Orthopedic Hospital Postdoctoral Innovation Practice Base The First Affiliated Hospital Jiangxi Medical College Nanchang University Nanchang 330006 China; ^4^ School of Materials Science and Engineering Stem Cells and Tissue Engineering Manufacture Center Hubei University Wuhan 430062 China

**Keywords:** ferroptosis, microneedle, MXene, nanozyme, wound healing

## Abstract

Reactive oxygen species (ROS) play a dual role in wound healing. They act as crucial signaling molecules and antimicrobial agents when present at moderate levels. However, excessive levels of ROS can hinder the healing process for individuals with diabetes. As a result, targeting ROS levels to maintain redox balance has become a promising strategy for improving wound recovery. Currently, no biomaterials have been reported to simultaneously up‐regulate and down‐regulate ROS to achieve broad‐spectrum antibacterial and antioxidant properties. Inspired by the site‐dependent effect of nanomaterials, a micron‐sized ferroferric oxide (Fe_3_O_4_)/MXene (FM) heterojunction is synthesized using a hydrothermal method. The FM heterojunction could scavenge extracellular ROS by activating catalase (CAT)‐like and superoxide dismutase (SOD)‐like nanozyme activities. Meanwhile, FM heterojunction could release ferric ions and ferrous ions by defect engineering to induce bacterial ferroptosis, up‐regulating intercellular ROS, and lipid peroxidation. For applications in vivo, FM heterojunction is incorporated into the tips of gelatin methacryloyl (GelMA)‐based microneedle (termed as GFM microneedle) using a two‐step casting technique. The results showed that GFM microneedle combined with photothermal therapy could improve *S. aureus*‐infected skin regeneration in diabetic rats. The effectiveness and safety of GFM microneedle are not less favorable than that of a commercial wound dressing. This study provides a proof‐of‐concept for heterojunction‐mediated regenerative medicine via a site‐dependent ROS‐targeting strategy.

## Graphical abstract

This study develops a micron‐sized ferroferric oxide (Fe_3_O_4_)/MXene (FM) heterojunction that scavenges extracellular ROS by activating catalase (CAT)‐like and superoxide dismutase (SOD)‐like activities, and releases ferric ions and ferrous ions to induce bacterial ferroptosis and combat bacterial infection. This study provides a proof‐of‐concept for heterojunction‐mediated regenerative medicine via a site‐dependent ROS‐targeting strategy.

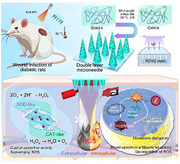

## 1 Introduction

Diabetic wounds are one of the most serious complications of diabetes. They are characterized by impaired healing due to several factors: bacterial infections, ulcer formation, tissue destruction, peripheral neuropathy, and compromised vascular function.^[^ ^1^, ^2^ ^]^ As the population ages, diabetic wounds have become a growing concern in healthcare worldwide.^[^ ^3^ ^]^ More than 25% of diabetic patients will suffer from diabetic wounds during their lifetime. Despite advancements in treatment methods such as hypoglycemia management, infection control, autologous skin transplantation, and negative pressure wound therapy (NPWT), the prognosis for diabetic wounds remains dismal.^[^ ^4^, ^5^ ^]^

Bioactive wound dressings, such as microneedle, hydrogel, electrospun nanofiber and 3D‐printed scaffold, are considered the next‐generation technique for skin regeneration.^[^ ^6^ ^]^ Microneedles are considered superior due to their well‐defined micro‐scale structure.^[^ ^7^ ^]^ Microneedles provide a minimally invasive method to penetrate the skin's dermis and deliver drugs or nanomaterials for targeted treatment. These microneedles can be made from biomolecules such as gelatin methacryloyl (GelMA), chitosan (CS), silk fibroin (SF), and hyaluronic acid (HA). They have the ability to absorb wound exudate, maintain a moist microenvironment, and enhance the adhesion, proliferation, and migration of host cells.^[^ ^8^, ^9^ ^]^ Currently, biomolecular microneedles hold promise for the future. Those innovative devices, which combine precise engineering with biological compatibility, offer numerous compelling advantages over traditional delivery methods and form an advanced multi‐functional therapeutic platform.

The primary requirements for diabetic wounds are broad‐spectrum antibacterial and antioxidant properties.^[^ ^10^ ^]^ Researchers have proposed various strategies to address this problem, including a reactive oxygen species (ROS)‐targeting strategy. Some nanomaterials, such as Fe_3_O_4_, molybdenum disulfide (MoS_2_), and copper sulfide (CuS), can up‐regulate ROS and exacerbate oxidative stress to kill bacteria.^[^ ^11^, ^12^, ^13^ ^]^ Other nanomaterials, such as cerium dioxide (CeO_2_) and manganese dioxide (MnO_2_), can down‐regulate ROS to promote skin regeneration.^[^ ^14^, ^15^, ^16^ ^]^ The ROS has a dual role in diabetic wound healing, particularly regarding its antibacterial and antioxidant properties. There is a significant challenge in developing a nanomaterial that can simultaneously up‐regulate and down‐regulate reactive oxygen species (ROS).

Nanomaterials entering cells is a sophisticated and intriguing process that affects their biomedical efficacy and other purposes. Some nanomaterials can penetrate cell membranes via endocytosis, receptor‐mediated uptake, or direct membrane penetration, whereas others meet biological limitations. Selective internalization can be engineered to create focused therapeutic approaches, not just a fault. The nanomaterial's physicochemical parameters (size, shape, surface charge, and surface modification), cell type, and physiological milieu affect internalization. Understanding and modifying these factors allows researchers to create smart nanomaterials that target certain cells for therapy or stay attached for diagnosis. Because cells absorb nanomaterials differently, specialized and effective therapeutic procedures can be developed.^[^ ^17^ ^]^ Inspired by this phenomenon, it was realized that the site‐dependent effect of nanomaterials can be applied to achieve opposite properties, including up‐regulation and down‐regulation of ROS. Fe_3_O_4_ nanoparticles are pH‐sensitive nanozymes that can scavenge excessive ROS by CAT‐like and SOD‐like activities under neutral and weakly alkaline conditions.^[^ ^18^, ^19^ ^]^ For example, Wei et al. reported a biomimetic anti‐oxidant system based on Fe_3_O_4_ nanoparticles and tannic acid (TA) for broad‐spectrum radical elimination.^[^ ^20^ ^]^ Fe_3_O_4_ nanoparticles can also release ferric ions and ferrous ions, which are transferred into bacterial cells, inducing bacterial ferroptosis and up‐regulating intercellular ROS.^[^ ^21^, ^22^ ^]^ In this study, it was assumed that Fe_3_O_4_ nanoparticles could regulate extracellular and intercellular ROS by activating a series of nanozyme‐like reactions and bacterial ferroptosis, respectively. To better accomplish this objective, Fe_3_O_4_ nanoparticles remain to be modified.

MXenes represent a novel family of 2D transition metal carbides, nitrides, and carbonitrides, which was first reported by Yury Gogotsi et al. in 2011.^[^ ^23^ ^]^ Among them, monolayer Ti_3_C_2_ nanosheets debut in a central position owing to their mature synthetic technique and rich advantages, such as high specific surface area, abundant termination groups, good electrical conductivity, and photothermal conversion efficiency.^[^ ^24^, ^25^ ^]^ The biocompatibility and biodegradability of Ti_3_C_2_ nanosheets are relatively good.^[^ ^26^ ^]^ In 2024, Deng et al. reported an MXene quantum dots (MQDs)/ferrous sulfide (FeS) heterojunction for effective combat against bacterial biofilm (BBF).^[^ ^27^ ^]^ The structure and biofunction of heterojunction are vital, as detailed in these reviews.^[^ ^28^, ^29^, ^30^ ^]^ Herein, an interdisciplinary concept of heterojunction was introduced to fabricate the Fe_3_O_4_/MXene (FM) heterojunction, which was supposed to reduce its band gap and activate the nanozyme‐like reactions. During the pre‐experiment, we also found that FM heterojunction could increase the release of ferric ions and ferrous ions, inducing bacterial ferroptosis. However, the potential mechanisms have rarely been investigated.

**Figure 1:**
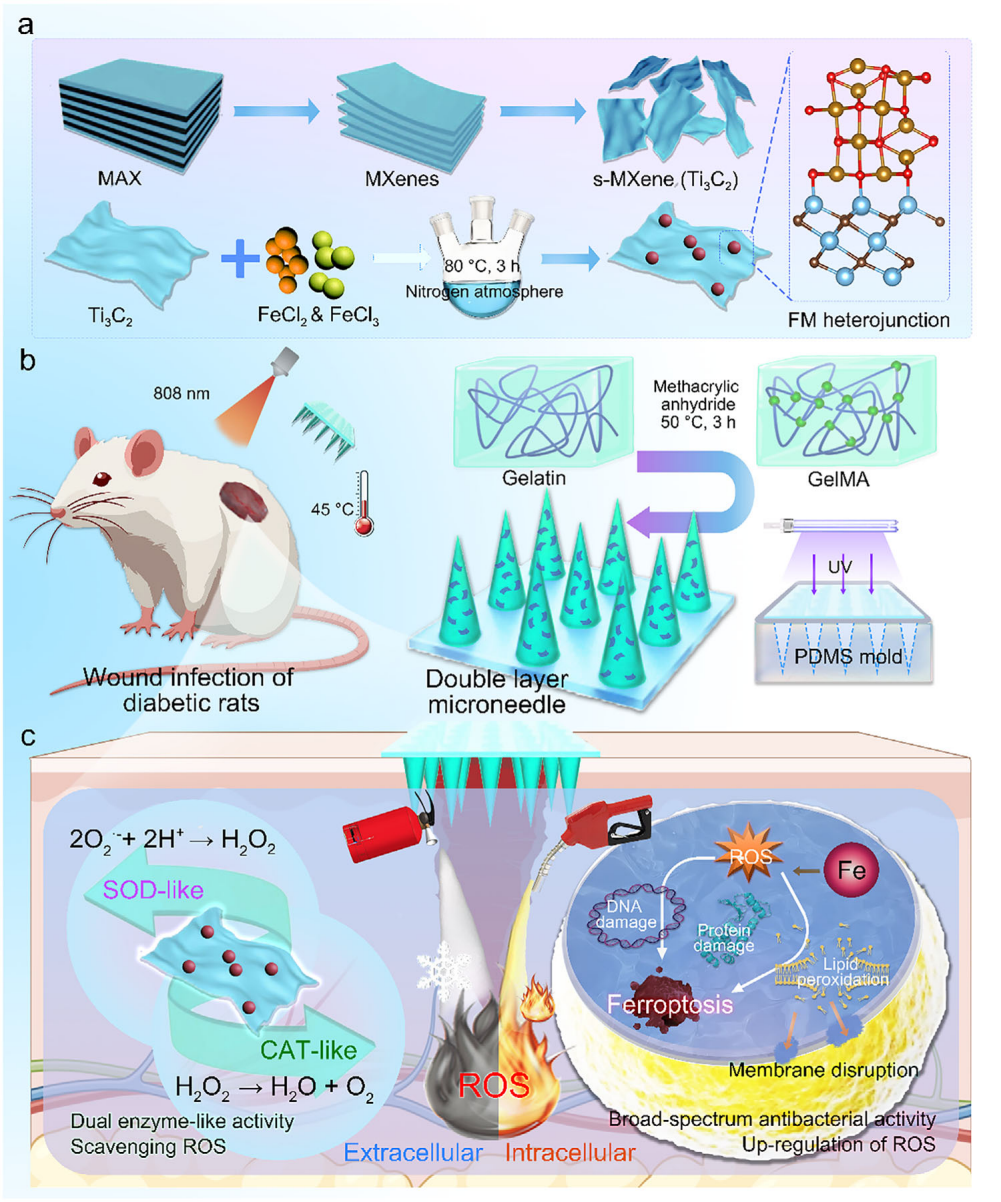
The preparation method and biomedical application of an FM heterojunction‐laden double‐layer biomolecular microneedle. a) FM heterojunction was prepared using a hydrothermal method; b) FM heterojunction was incorporated into the tips of GelMA microneedle for the treatment of diabetic wound; c) The microneedle could accelerate wound healing by simultaneously up‐regulating intercellular ROS and down‐regulating extracellular ROS. The potential mechanisms are highlighted.

A diagram of this study is shown in **Figure** **1**. The FM heterojunction was first synthesized using a hydrothermal method and then immobilized onto the needle tips of the GelMA microneedle using a two‐step casting technique. The obtained double‐layer microneedle was termed a GFM microneedle. In this study, we will comprehensively characterize the physiochemical properties, photothermal conversion ability, antibacterial properties, and antioxidative activities. In particular, the mechanisms of antibacterial and anti‐oxidative activities are emphasized, focusing on nanozyme‐like reactions and bacterial ferroptosis. This study is significantly innovative in exploring the interactions between the formation of vacancy defects, the release of ferric ions and ferrous ions, and bacterial ferroptosis. The products of this study will provide an effective and safe biomaterial for the precision treatment of diabetic wounds.

## 2 Results and Discussion

### 2.1 Characterizations of FM Heterojunction

#### 2.1.1 FM Heterojunction was Successfully Synthesized

**Figure 2:**
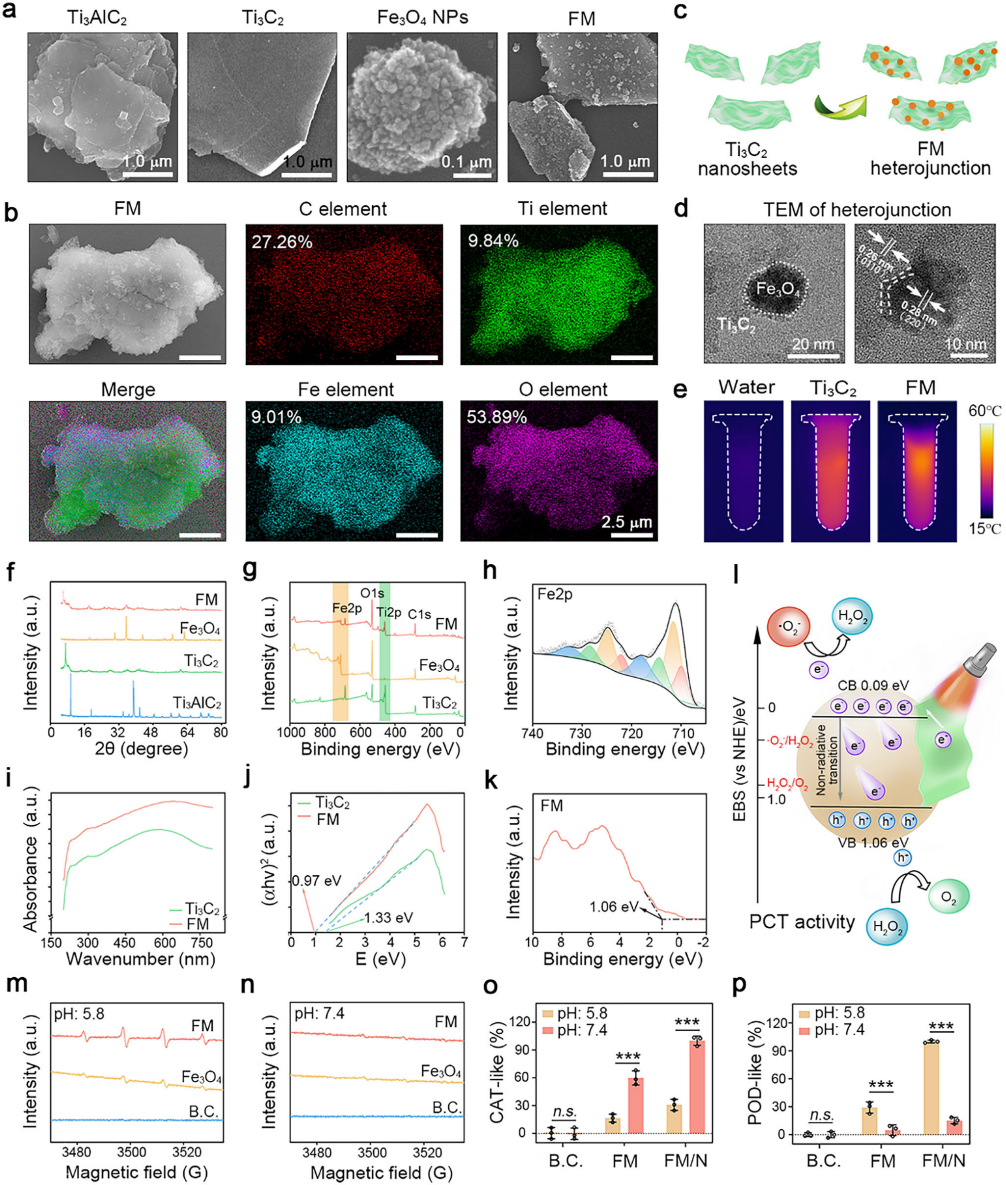
The physiochemical characterizations of FM heterojunction. a) SEM images of Ti_3_AlC_2_, Ti_3_C_2_, Fe_3_O_4,_ and FM. Scale bar: 1 µm or 0.1 µm; b) SEM mapping of FM heterojunction. Scale bar: 2.5 µm; c) The structure of FM heterojunction; d) TEM images. Scale bar: 20 or 10 nm; e) Photothermal conversion test in vitro; f) XRD spectrum; g,h) XPS spectrum; i) UV–vis spectrum; j) The bandgap calculated by Tauc‐Plot method; k) XPS‐VB spectrum; l) The potential anti‐oxidative mechanism; m) EPR test for detecting hydroxyl radical (·OH) at pH value of 5.8; n) EPR test at pH value of 7.4; o,p) Evaluations of CAT‐like and POD‐like activities (n = 3). Values are expressed as the mean ± SD, *n.s*. indicates no significance, ^***^ *P* < 0.001.

Bulk Ti_3_AlC_2_ was processed into monolayer Ti_3_C_2_ nanosheets using an improved etching method. Hydrofluoric acid (HF) and lithium chloride (LiCl) as the intercalation agents replaced the aluminum (Al) layer of bulk Ti_3_AlC_2_ to obtain the organ‐shaped product. It was subsequently dispersed into monolayer Ti_3_C_2_ nanosheets by ultrasonic oscillation. As shown in **Figure** **2**a, the specific surface area of monolayer Ti_3_C_2_ nanosheets significantly increased when compared to bulk Ti_3_AlC_2_, which provides sufficient sites for chemical modification. Subsequently, FM heterojunction was synthesized using a hydrothermal reaction. Fe_3_O_4_ nanoparticles are evenly distributed on FM's surface. An SEM‐EDS mapping was performed to visualize the element content. As shown in Figure 2b, the content of the characteristic Fe element was 9.01%, confirming that Fe_3_O_4_ nanoparticles were successfully immobilized onto the FM. Figure 2c shows the nanostructure of FM. It was inferred that Ti_3_C_2_ nanosheets and Fe_3_O_4_ nanoparticles are combined into one system to form the heterojunction. TEM was performed to verify this hypothesis (Figure 2d). The interplanar spacing of Ti_3_C_2_ nanosheets and Fe_3_O_4_ nanoparticles was 0.26 and 0.28 nm, respectively. Meanwhile, a transitional signal could be found in their interfaces. In conclusion, FM heterojunction was successfully synthesized. TEM‐EDS mapping was also performed to visualize the element content of FM heterojunction (Figure S1, Supporting Information). The obtained results were consistent with those in Figure 2b.

#### 2.1.2 FM Heterojunction Exhibited Good Photothermal Nanozyme‐Like Potential

As shown in Figure 2e and Figure S2 (Supporting Information), the photothermal effect of FM heterojunction was investigated. Under NIR irradiation, the FM solution heated up faster than the Ti_3_C_2_ solution of the same concentration, indicating better photothermal conversion efficiency. This phenomenon could be attributed to the incorporation of Fe_3_O_4_ nanoparticles, which are ideal photothermal agents.^[^ ^31^ ^]^ As shown in Figure S3 (Supporting Information), Fe_3_O_4_ nanoparticles could also endow the FM heterojunction with enhanced dispersion and water stability.

Figure 2f and Figure S4 (Supporting Information) show the XRD spectrum of FM heterojunction. Compared to bulk Ti_3_AlC_2_, the characteristic peak (104) of the Al layer at 38.8° disappeared in the Ti_3_C_2_ group. Meanwhile, the characteristic peak (002) of Ti_3_C_2_ nanosheets shifted from 9.6° to 7.1°. These results are consistent with previous reports, indicating that monolayer Ti_3_C_2_ nanosheets were successfully prepared.^[^ ^32^ ^]^ The characteristic peaks of Fe_3_O_4_ nanoparticles, including (111), (222), and (440), are consistent with the previous reports.^[^ ^33^ ^]^ FM heterojunction exhibited a characteristic peak of Ti_3_C_2_ nanosheets (002) and three characteristic peaks of Fe_3_O_4_ nanoparticles. Thus, it could be safely concluded that Fe_3_O_4_ nanoparticles were immobilized onto the surface of Ti_3_C_2_ nanosheets.

Figure 2g,h and Figure S5 (Supporting Information) show the XPS spectrum of FM heterojunction. Ti_3_C_2_ nanosheets exhibited a characteristic peak of Ti 2p located at 459 eV, and Fe_3_O_4_ nanoparticles exhibited a distinct peak of Fe 2p located at 720 eV. FM heterojunction exhibited four characteristic peaks, including Fe 2p at 720 eV, O 1s at 532 eV, Ti 2p at 459 eV, and C 1s at 285 eV. The Fe 2p spectrum of FM heterojunction exhibited four characteristic peaks, including Fe^3+^ 2p₁/₂ at 724 eV, Fe^3+^ 2p₃/₂ at 710 eV, Fe^2+^ 2p₁/₂ at 720 eV, Fe^2+^ 2p₃/₂ at 709 eV. These results further confirmed that FM heterojunction was composed of Fe_3_O_4_ nanoparticles and Ti_3_C_2_ nanosheets.

As shown in Figure 2i,j, the bandgap was detected by the UV–vis spectrum. It was 0.97 eV for FM heterojunction and 1.33 eV for Ti_3_C_2_ nanosheets, respectively. The smaller the bandgap, the easier it is for electrons to be excited from the valence band to the conduction band.^[^ ^34^ ^]^ As shown in Figure S6a–c (Supporting Information), electrochemical impedance test, linear sweep voltammetry test, and transient photocurrent curve test were performed to characterize the photocatalytic activity of FM heterojunction. Compared to Ti_3_C_2_ nanosheets, the Nyquist semicircle diameter of FM heterojunction was significantly narrowed, and the slope of the linear sweep voltammetry curve was increased. These results indicated that the interface of FM heterojunction has the least charge transfer resistance, the largest conductivity, and the fastest electron transfer speed. Through the transient photocurrent curve, it could be found that under NIR irradiation, the photo‐response current density of FM heterojunction was significantly increased due to the excellent electron‐hole pair separation efficiency. Thus, it was concluded that the photocatalytic performance of FM heterojunction was improved considerably.

The valence band of FM heterojunction was 1.06 eV (Figure 2k). The PL spectrum is shown in Figure S7 (Supporting Information). FM heterojunction showed a decrease in excitation light among a wavelength of 600–700 nm, indicating a reduction in recombination but an increase in separation efficiency of electron‐hole pairs. A band diagram of FM heterojunction was made. As shown in Figure 2l, FM heterojunction can generate photoelectron‐hole pairs under NIR irradiation. The photoelectron (e^−^) reacts with superoxide anion (O_2_^·−^) to produce hydrogen peroxide (H_2_O_2_). After that, hydrogen peroxide is cleared by hole (h^+^) to produce non‐toxic water and oxygen. A series of EPR analyses (Figure 2m,n) and biochemical tests (Figure 2o,p; Figure S8a,b, Supporting Information) were performed. It was proved that FM heterojunction has pH‐dependent CAT‐like and POD‐like activities. As shown in Figure S9 (Supporting Information), the CAT‐like activity also exhibited a typical NIR‐dependent effect.

The characteristics of diabetic wounds include but are not limited to dynamically changing pH value, overactivated oxidative stress, and chronic bacterial infection.^[^ ^35^ ^]^ Affected by the leakage of micro‐vessels, the pH value of unhealed diabetic wounds fluctuates between 7–9.^[^ ^36^ ^]^ However, the microenvironment of healthy skin is acidic, with a pH value ranging from 4–6. The abnormal pH value can strongly intervene in a series of intercellular processes (enzyme activity, macromolecular synthesis, metabolite transport) and tissue regeneration processes (collagen formation and angiogenesis), delaying wound healing.^[^ ^37^ ^]^

The pH‐dependent CAT‐like and POD‐like activities of FM heterojunction are highly matched with the clinical needs of diabetic wounds. Under neutral and weakly alkaline conditions, FM heterojunction can remove ROS, alleviate oxidative stress, remodel the wound microenvironment, and promote tissue regeneration. After wound healing, the anti‐oxidative effect of FM heterojunction disappears and shows good biocompatibility. Notably, FM heterojunction also exhibited good broad‐spectrum antibacterial activity by a series of nanozyme‐independent molecular mechanisms. This issue will be discussed in the subsequent chapters.

### 2.2 Characterizations of FM‐Laden Composite Materials

#### 2.2.1 FM‐Laden GelMA Hydrogel was Successfully Prepared

FM heterojunction is not suitable for use alone as a wound dressing. Currently, the application is still facing several challenges, such as single performance, insufficient tissue delivery, potential adhesion to tissue, and non‐degradability. In previous works,^[^ ^38^, ^39^, ^40^ ^]^ our group has reported a series of GelMA hydrogel‐based microneedles. GelMA hydrogel can absorb the wound exudates, maintain a moist environment, and provide vital cues for guiding cell migration and proliferation. The microneedle tips can also be used to deliver nanomaterials with a smaller dosage of administration. Herein, it was assumed that FM heterojunction and GelMA hydrogel‐based microneedles could learn from other's strong points and close the gap.

**Figure 3:**
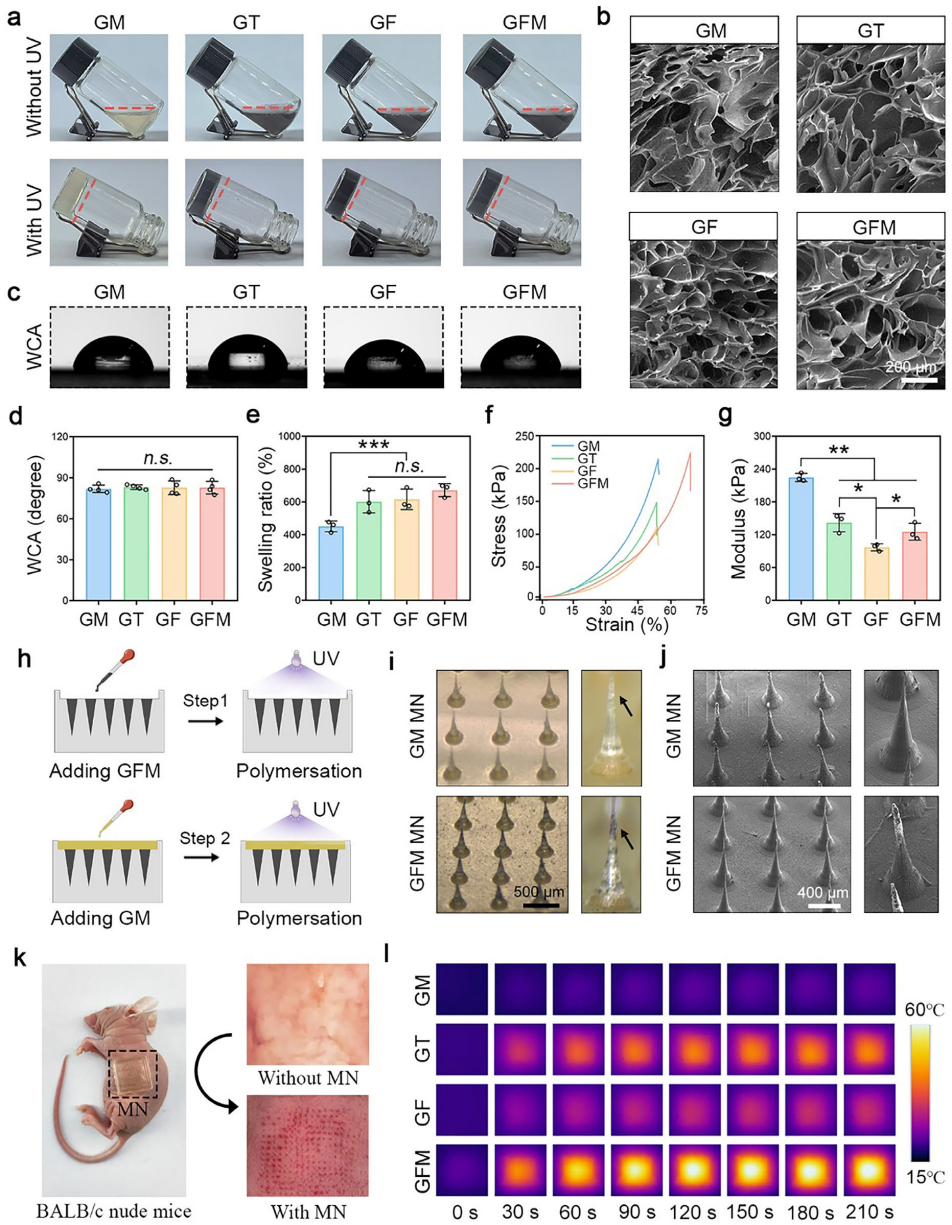
The preparation and characterizations of FM‐laden hydrogels and microneedles. a) Optical images of neat GelMA hydrogel (GM), GelMA/Ti_3_C_2_ hydrogel (GT), GelMA/Fe_3_O_4_ hydrogel (GF), and GelMA/FM heterojunction hydrogel (GFM) before and after UV crosslinking; b) SEM images of freeze‐dried hydrogels. Scale bar: 200 µm; c) Optical images of water contact angle (WCA); d) Quantitative results of WCA (n = 4); e) Equilibrium swelling ratio (n = 3); f) Stress–strain curves (n = 1); g) Compressive modulus (n = 3); h) A diagram showing the preparation process of double‐layer microneedles; i) Optical images of microneedles. Scale bar: 500 µm; j) SEM images. Scale bar: 400 µm; k) GFM microneedle punctured into the skin of BALB/c nude mice; l) Photothermal conversion test of the microneedles in vitro; Values are expressed as the mean ± SD, *n.s*. indicates no significance, ^*^ *P* < 0.05, ^**^ *P* < 0.01, ^***^ *P* < 0.001.

As shown in **Figure** **3**a, a series of composite hydrogels were prepared by ultraviolet (UV) crosslinking. GelMA served as the molecular skeleton to provide adequate mechanical support.^[^ ^41^ ^]^ The nanomaterials were evenly dispersed into the network. These hydrogels were freeze‐dried and exhibited a typical porous structure (Figure 3b). Compared to GM hydrogel, the incorporation of FM heterojunction did not change the hydrophilicity of GFM hydrogel (Figure 3c,d). Meanwhile, GFM hydrogel exhibited enhanced swelling ability and less mechanical strength (Figure 3e–g). Significant differences were observed between GM and GFM groups (*P* < 0.01).

#### 2.2.2 The Hydrogels were Processed into Double‐Layer Microneedles

As shown in Figure 3h, a two‐step casting method was used to fabricate the double‐layer microneedles. The base layer was composed of neat GM hydrogel, and the tip layer was composed of bioactive composite hydrogels. As shown in Figure 3i,j and Figure S10 (Supporting Information), GM and GFM microneedles with typical tip arrays were successfully obtained. They could easily puncture into the skin to deliver FM heterojunction and other nanomaterials (Figure 3k). These microneedles had good photothermal conversion ability (Figure 3l; Figure S11a, Supporting Information). Among them, the GFM microneedle exhibited the best photothermal efficiency. GFM microneedle could be used for on‐demand photothermal therapy by modulating the power of NIR light (Figure S11b, Supporting Information). The photothermal stability of the GFM microneedle could meet the general requirements (Figure S11c, Supporting Information).

As shown in Figure S12 (Supporting Information), a degradation assay of collagenase was performed. On day 7, the degradation rate was 31.21 ± 5.17% for GM microneedle and 25.51 ± 3.92% for GFM microneedle; on day 14, the degradation rate was 89.40 ± 2.78% for GM microneedle and 86.96 ± 2.30% for GFM microneedle. The wound‐healing process lasted for ≈16 days. The microneedles were almost degraded without hindering wound healing.

### 2.3 GFM Microneedle Rescued ROS by Activating Cascading Nanozyme

#### 2.3.1 FM Heterojunction Endowed GFM Microneedle with Photothermal Nanozyme‐Like Activities

In the previous chapter, we have demonstrated that FM heterojunction has anti‐oxidative potential under neutral and weakly alkaline conditions. FM heterojunction was immobilized into a GFM microneedle without chemical modifications. Thus, the incorporation of FM heterojunction is supposed to endow the GFM microneedle with enhanced anti‐oxidative ability. Herein, the photothermal nanozyme‐like activities of the GFM microneedle were comprehensively characterized.

**Figure 4:**
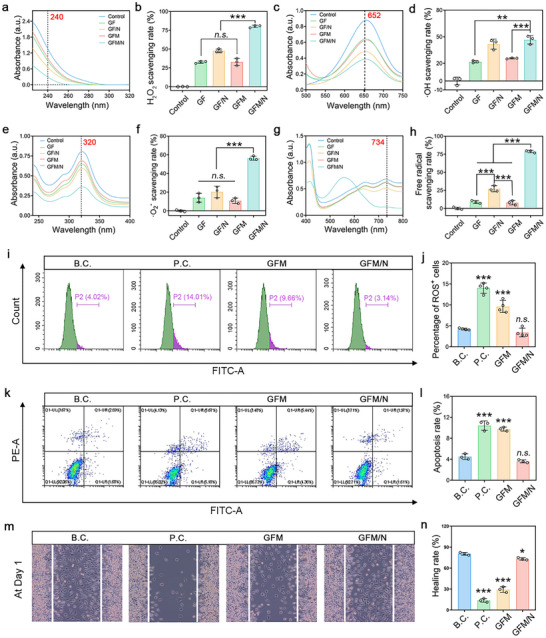
Evaluations of anti‐oxidative activity and application of GFM microneedle. a,b) H_2_O_2_ scavenging activity (n = 3); c,d)·OH scavenging activity (n = 3); e,f) O_2_^·−^ scavenging activity (n = 3); g,h) Free radical scavenging activity (n = 3); i,j) Flow cytometry for detecting intracellular ROS (n = 4); k,l) Flow cytometry for detecting cell apoptosis (n = 3); m,n) Scratching assay for detecting cell migration (n = 3). Values are expressed as the mean ± SD, *n.s*. indicates no significance, ^**^ *P* < 0.01. ^***^ *P* < 0.001.

As shown in **Figure** **4**a,b, the GFM microneedle exhibited good H_2_O_2_ scavenging activity. It could be significantly improved with the help of photothermal therapy (GFM/N group *v.s*. GFM group, *P* < 0.001). Meanwhile, the H_2_O_2_ scavenging activity of the GFM/N group was significantly better than that of the GF/N group, showing excellent superiority compared to traditional nanozymes (Fe_3_O_4_ nanoparticles). According to our results, the potential mechanisms of this phenomenon include but are not limited to smaller bandgap and better photothermal efficiency.

As shown in Figure 4c–h, the GFM/N group also exhibited the best ·OH scavenging activity, O_2_ **^·−^** scavenging activity, and free radical scavenging activity among the five groups. Significant differences could be found between the GFM/N group and other groups (*P* < 0.01). Thus, we inferred that GFM microneedle combined with photothermal therapy (GFM/N) is highly desirable for anti‐oxidative biomedical applications.^[^ ^18^, ^42^ ^]^ Notably, the anti‐oxidative property of the GFM microneedle could be summarized into a cascading nanozyme, which is presented in Figure S13 (Supporting Information).

#### 2.3.2 GFM Microneedle Rescued Oxidative Stress of Diabetic Wounds

In diabetic wounds, the overactivation of reactive oxygen species (ROS) due to the hyperglycemic microenvironment causes oxidative stress in various biomacromolecules and cell membranes. This situation further leads to prolonged inflammation, delays in angiogenesis, and hinders the wound healing process of the wound.^[^ ^43^, ^44^ ^]^ GFM microneedle is expected to rescue oxidative stress of diabetic wounds by activating cascading nanozyme. Herein, an H_2_O_2_‐induced cell model of oxidative stress was established to verify this assumption.

As shown in Figure 4i,j, the positive control (P.C.) group was treated with 50 µM of H_2_O_2_ for 48 h, and the percentage of ROS^+^ cells increased from 4.22 ± 0.21% to 13.99 ± 1.22%. This phenomenon suggested that the cell model was successfully established. The percentage of ROS^+^ cells was 9.61 ± 1.43% for the GFM group and 3.41 ± 1.04% for the GFM/N group, respectively. No significant difference was observed between the blank control (B.C.) group and the GFM/N group (*P* > 0.05). These results indicated that GFM/N could protect these cells from H_2_O_2_‐induced oxidative stress. Furthermore, GFM/N could also inhibit cell apoptosis and promote cell migration, showing potential beneficial effects for tissue regeneration (Figure 4k–n).

The above bioactivities of GFM/N are mainly attributed to FM heterojunction, which is physically immobilized into GFM microneedle. FM heterojunction cannot be internalized by host cells owing to the spatial separation of the hydrogel matrix. Thus, the site of action of FM heterojunction is located outside the cells rather than inside the cells. FM heterojunction and GFM/N can rescue extracellular ROS but cannot rescue intercellular ROS. This conclusion is very interesting because intercellular ROS can be individually upregulated and explored for other biomedical applications like antibacterial therapy. Currently, it is challenging for nanomaterials to downregulate extracellular ROS but upregulate intercellular ROS at the same time. The underlying mechanism has been rarely revealed.

### 2.4 GFM Microneedle Combated Infection by Inducing Bacterial Ferroptosis

#### 2.4.1 GFM/N was Broad‐Spectrum Antibacterial

**Figure 5:**
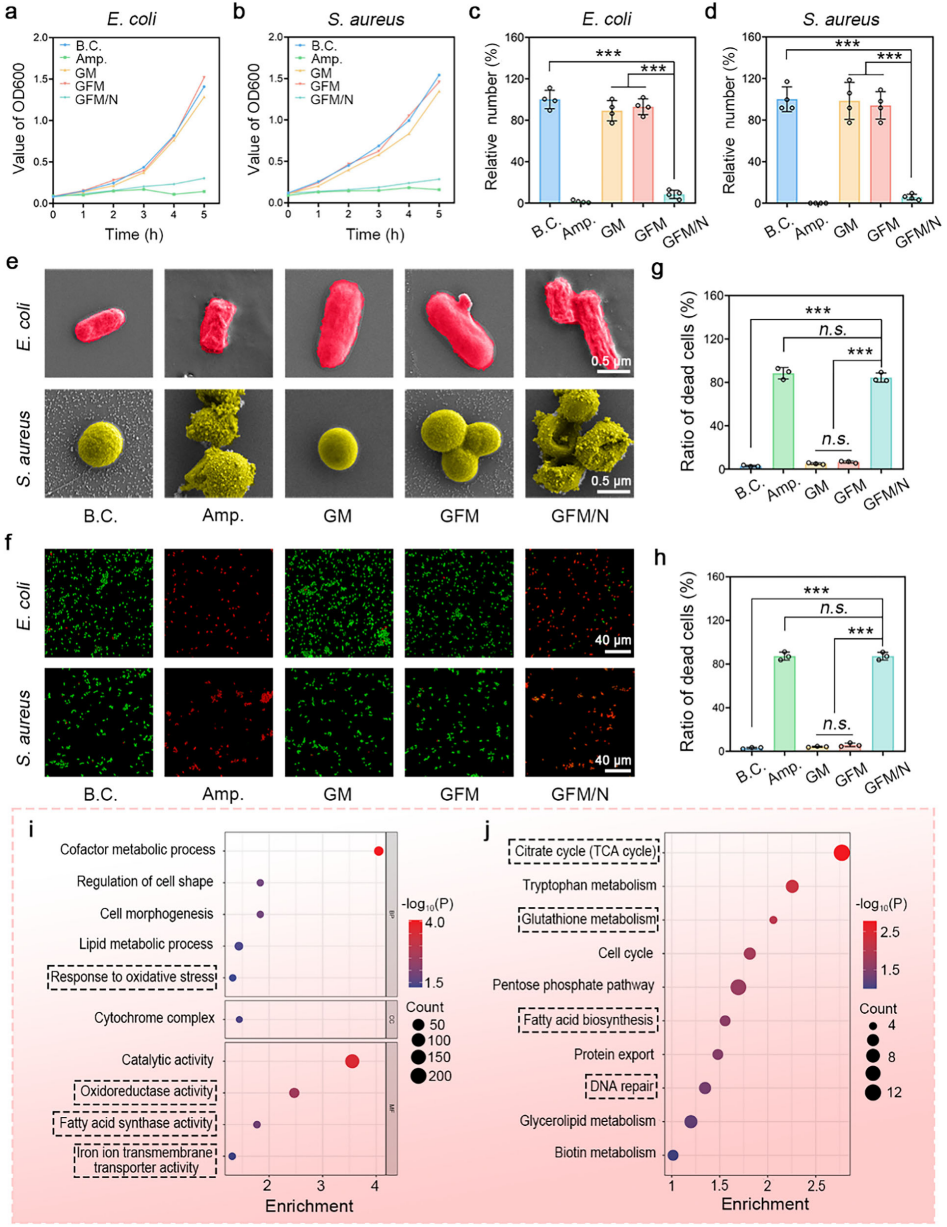
Evaluations of broad‐spectrum antibacterial activity of GFM microneedle. a,b) Bacterial proliferation curves of *E. coli* and *S. aureus*; c,d) Relative number of bacterial clones of *E. coli* and *S. aureus* (n = 4); e) SEM images of bacteria. Scale bar: 0.5 µm; f) Live/dead bacterial staining images. Scale bar: 40 µm; g,h) Ratio of dead bacterial cells per field of *E. coli* and *S. aureus* (n = 3). i,j) GO and KEGG analysis of up‐regulated DEGs. Values are expressed as the mean ± SD, *n.s*. indicates no significance, ^***^ *P* < 0.001.

In this chapter, the broad‐spectrum antibacterial activity of GFM microneedle was evaluated. The results of the bacterial proliferation assay are shown in **Figure** **5**a,b. GFM/N could effectively inhibit the proliferation ability of both *E. coli* and *S. aureus*, but GFM could not. The anti‐proliferation effect of GFM/N was comparable to that of ampicillin (Amp. group). The results of the clone formation assay are shown in Figure S14 (Supporting Information) and Figure 5c,d. GFM/N could effectively inhibit bacterial survival ability. Compared to the B.C. group, a significant difference was observed (*P* < 0.001). Notably, GFM/N could also inhibit the proliferation and survival of a drug‐resistant bacterial, namely MRSA, indicating good broad‐spectrum antibacterial activity (Figure S15, Supporting Information).

The treated bacteria were dehydrated. As shown in Figure 5e, the cells in the GFM group underwent mild morphological contraction, while those in the Amp. and GFM/N groups underwent severe morphological contraction. The morphology of bacteria can be determined by various physicochemical factors. ^[^ ^45^, ^46^ ^]^Usually, bacterial morphology is more typical under suitable conditions. When there are substances in the environment that are not suitable for bacterial growth (such as drugs, antibiotics, and antibodies), bacteria often exhibit irregular shapes, known as pleomorphism, pear‐shaped, balloon‐shaped, filamentous, etc. Our results could reflect the broad‐spectrum antibacterial effect of GFM/N, which is consistent with those in Figure 5a–d.

As shown in Figure 5f–h, a live/dead bacterial staining assay was performed to visualize the bacterial viability. Live bacteria were dyed green, and dead bacteria were dyed red. It was observed that most bacteria cells in Amp. and GFM/N groups were dye red, showing excellent antibacterial effect. For *E. coli*, the ratio of dead cells was 4.48 ± 0.69% for the B.C. group, 84.87 ± 3.15% for Amp. group, 4.18 ± 1.4% for GM group, 5.65 ±1.28% for GFM group, and 85.65 ± 2.77% for GFM/N group; For *S. aureus*, similar results were obtained. No significant difference was observed between the Amp. group and the GFM/N group (*P* > 0.05). Based on these results, it could be concluded that the broad‐spectrum antibacterial activity of GFM/N is comparable to that of ampicillin.

#### 2.4.2 GFM/N Could Induce Bacterial Ferroptosis by Releasing Fe^2+^/Fe^3+^ Ions

**Figure 6:**
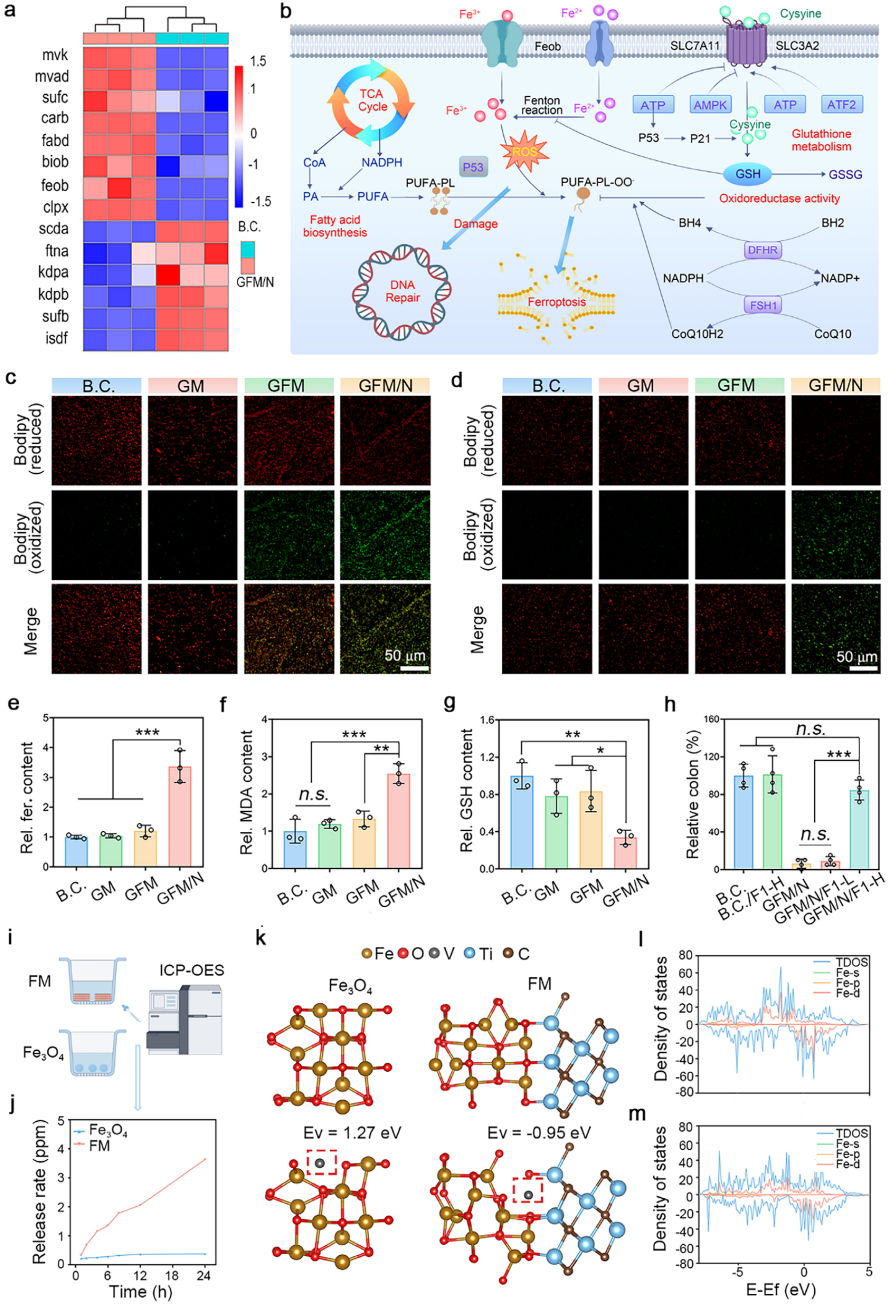
Ferroptosis contributed to the antibacterial mechanism of GFM microneedle. a) Heatmap of top‐14 DEGs related to ferroptosis; b) A diagram showing the potential mechanisms of ferroptosis; c) Ferroptosis state of *E. coli* was labeled using an LPO staining kit. Scale bar: 50 µm; d) Ferroptosis state of *S. aureus* was labeled. Scale bar: 50 µm; e–g) Relative content of ferrous iron, MDA, and GSH in *S. aureus* (n = 3); h) Rescue assay by ferroptosis inhibitor; i,j) The releasing dynamic of Fe^2+^/Fe^3+^ ions was detected by ICP‐OES; k–n) DFT analysis of vacancy defect of FM heterojunction. Values are expressed as the mean ± SD, *n.s*. indicates no significance, ^*^ *P* < 0.05, ^**^ *P* < 0.01, ^***^ *P* < 0.001.

A prokaryotic transcriptome sequencing was performed. Two groups, including the blank control group and the GFM/N group, were set, and three independent samples were used for statistical analysis. As shown in Figure S16a (Supporting Information), 608 down‐regulated genes and 561 up‐regulated genes were screened out. The relative expression of these differentially expressed genes (DEGs) was shown in a heatmap (Figure S16b, Supporting Information). Figure 5i shows the GO analysis of up‐regulated DEGs. The up‐regulated DEGs were enriched in such biological processes and molecular functions, including response to oxidative stress, oxidoreduction activity, fatty acid synthase activity, and iron ion transmembrane transporter activity. Figure 5j shows the KEGG analysis of up‐regulated DEGs. TCA cycle, glutathione metabolism, fatty acid biosynthesis, and DNA repair were also enriched. The GO and KEGG analysis of down‐regulated DEGs is shown in Figure S17 (Supporting Information). These results reflected the antibacterial mechanisms of GFM/N. As shown in **Figure** **6**a, fourteen DEGs related to ferroptosis were screened out. Ferroptosis is a novel antibacterial approach that has recently attracted much attention.^[^ ^47^, ^48^ ^]^ Based on the available knowledge, ferroptosis is highly associated with the results of GO and KEGG analysis in Figure 5i,j. Their potential interactions are summarized and exhibited in Figure 6b. It was assumed that GFM/N could induce bacterial ferroptosis, which represents one of the most important mechanisms of broad‐spectrum antibacterial activities.

Lipid peroxidation (LPO) induced by intercellular ROS is one of the markers of ferroptosis.^[^ ^48^ ^]^ As shown in Figure 6c,d, a bacterial LPO staining assay was performed. The bacterial cells in the oxidation state were dyed green, and those in the reduction state were dyed red. Compared to the B.C. group, most bacterial cells of *E. coli* and *S. aureus* in the GFM/N group were brighter in green, indicating an overactivated ferroptosis state. As shown in Figure 6e–g, GFM/N could not only increase the content of intercellular ferrous ion and malondialdehyde (MDA) but also decrease the content of anti‐oxidative glutathione (GSH). The phenomenon suggested that GFM/N could upregulate intercellular ROS and induce LPO and bacterial ferroptosis. Ferrostatin‐1 (F‐1) is a selective inhibitor of ferroptosis.^[^ ^49^ ^]^ As shown in Figure 6h and Figure S18 (Supporting Information), the incorporation of F‐1 with high dosage (GFM/N/F1‐H group) could effectively rescue the anti‐survival activity of GFM/N. Thus, the antibacterial role of GFM/N‐mediated ferroptosis was solid. Ferroptosis is a Fe^2+^/Fe^3+^‐dependent death model.^[^ ^50^, ^51^ ^]^ Fe^2+^/Fe^3+^ ions can react with ethylene diamine tetra‐acetic acid (EDTA) to form insoluble complexes. As shown in Figure S19a,b (Supporting Information), the incorporation of EDTA could effectively rescue the anti‐proliferation activity of GFM/N. It could be concluded that the Fe^2+^/Fe^3+^ ions released by GFM/N could activate bacterial ferroptosis and eventually lead to bacteria death.

#### 2.4.3 FM Heterojunction's Vacancy Defect Determined the Fe^2+^/Fe^3+^ Ions Release

As shown in Figure 6i, the releasing dynamics of Fe^2+^/Fe^3+^ ions were detected by ICP‐OES. Compared to Fe_3_O_4_ nanoparticles, FM heterojunction exhibited a faster‐releasing rate (Figure 6j). This phenomenon might be attributed to the vacancy defect of F_3_O_4_ formed during the preparation process of FM heterojunction.^[^ ^52^ ^]^ As shown in Figure S20 (Supporting Information), the ratio of Fe^2+^/Fe^3+^ ions was 29.3: 70.7. A DFT analysis was performed to verify this assumption. As shown in Figure 6k, the geometric optimization models of Fe_3_O_4_ nanoparticles and FM heterojunction were constructed. It was assumed that iron vacancies in Fe_3_O_4_ nanoparticles are located on the crystal surface, and iron vacancies in FM heterojunction are located at the heterojunction interface. We optimized the structures and found that the formation energy (Ev) of iron vacancies in Fe_3_O_4_ nanoparticles was 1.27 eV and that in FM heterojunction was −0.95 eV. These results indicated that the structure of Fe_3_O_4_ nanoparticles is more stable, and the FM heterojunction makes it easier to form iron vacancies and release iron ions. As shown in Figure 6l,m, the total density of states (TDOS) and density of states (DOS) of FM heterojunction with and without iron vacancies were calculated. The results showed that FM heterojunction with iron vacancies exhibited a continuous and distinct Fe d orbital state near the Fermi level, which made its electron transfer efficiency higher. Under near‐infrared light irradiation, electron transfer was more likely to occur in FM heterojunction, clearing reactive oxygen species.

### 2.5 GFM Microneedle Accelerated Diabetic Wound Healing

**Figure 7:**
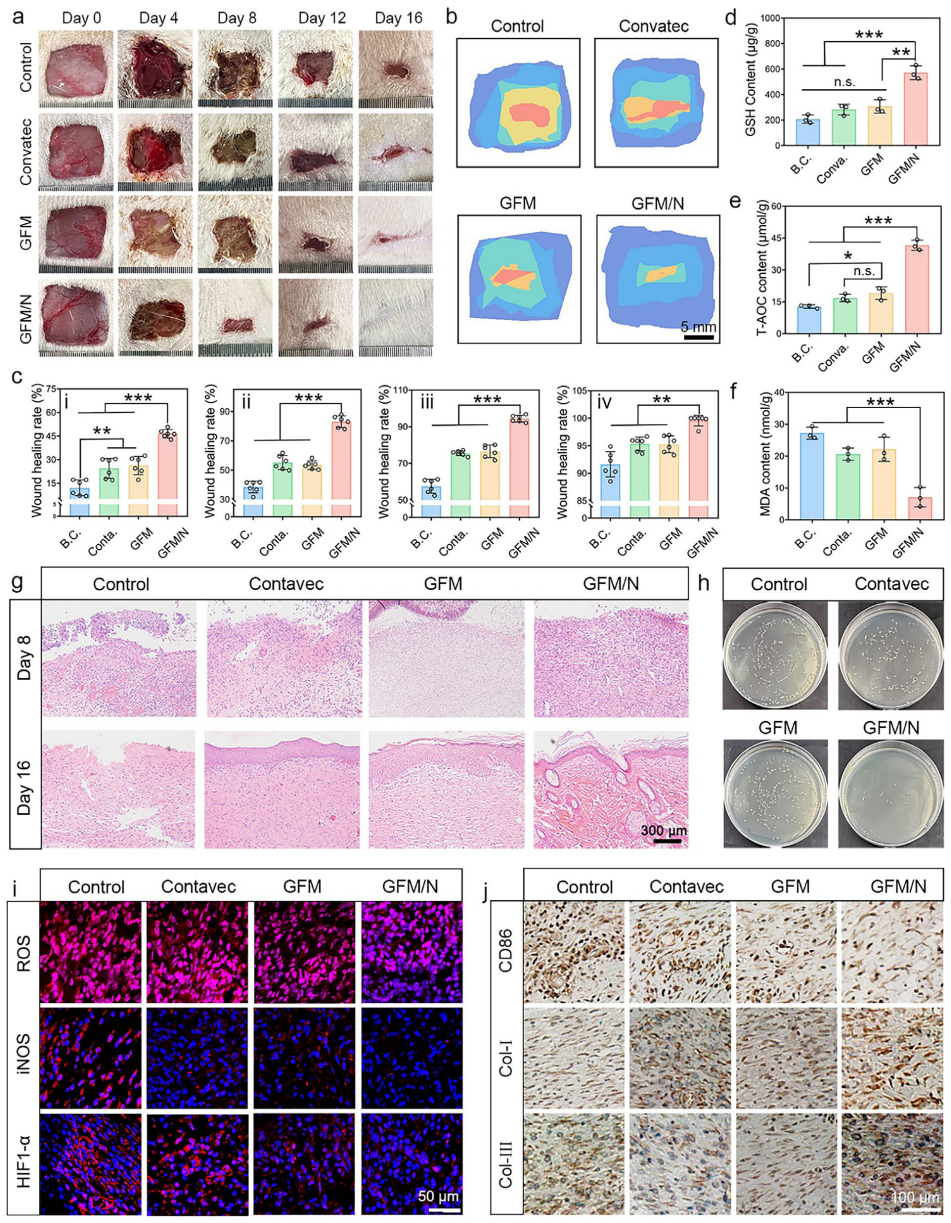
GFM microneedle combined with PTT significantly improved diabetic wound healing. a) Optical images of wound sites; b) Traces of wound healing process. Scale bar: 5 mm; c) Quantitative results of wound healing rate at each time point (n = 6); d–f) The content of GSH, T‐AOC, and MDA in neo‐skin tissue (n = 3); h) Antibacterial evaluation in vivo; i) Immunofluorescence staining images of neo‐skin tissue at day 8. Scale bar: 50 µm; j) Immunohistochemical staining images of neo‐skin tissue at day 16. Scale bar: 100 µm. Values are expressed as the mean ± SD, *n.s*. indicates no significance, ^*^ *P* < 0.05, ^**^ *P* < 0.01, ^***^ *P* < 0.001.

In this study, an STZ‐induced diabetic model of SD rats was established to evaluate the application effect of GFM microneedle. A square‐thickness skin defect with a side length of 15 mm was created in each animal and immediately infected with *S. aureus*. As shown in **Figure** **7**a, the wounds were treated with GFM microneedle, GFM microneedle combined with photothermal therapy (GFM/N), and commercial antibacterial dressing from Contavec, respectively. The B.C. group was not treated. The wounds in each group almost healed within 16 days. The wound healing dynamics were traced and shown in Figure 7b. It could be found that the GFM/N group healed the fastest among the four groups. Figure 7c shows the quantitative results of the wound healing rate. At varied time points, significant differences could be observed between the GFM/N group and other groups (*P* < 0.01). In conclusion, GFM/N could effectively accelerate diabetic wound healing.

On Day 8, neo‐skin tissue was resected and detected by a series of biochemical tests (Figure 7d–f). Compared to the B.C. group, the GFM/N group exhibited up‐regulated content of anti‐oxidative GSH and T‐AOC and down‐regulated content of pro‐oxidative MDA (*P* < 0.001). These results indicated that GFM/N treatment played an anti‐oxidative role in the early stage of wound healing. Neo‐skin tissue was also collected for H&E staining assay (Figure 7g). On Day 8, the GFM/N group exhibited less inflammatory cell infiltration, more blood vessels, and granulation tissue. On Day 16, the GFM/N group exhibited complete keratinized epithelium and neo‐hair follicle, which were not found in other groups. These results indicated that histological regeneration of diabetic wounds was largely improved. Notably, the pro‐regenerative ability of GFM/N could also be attributed to its antibacterial activity, which has been verified in vivo (Figure 7h; Figure S21, Supporting Information).

Figure 7i shows the immunofluorescence staining images on Day 8, and the quantitative results are shown in Figure S22a–c (Supporting Information). GFM/N could significantly clear the ROS in situ and relieve the hypoxia of diabetic wounds by down‐regulating the expression of HIF‐1α.^[^ ^53^ ^]^ As shown in Figure S23a–c (Supporting Information), GFM/N could also inhibit tissue inflammation (labeled by CD45) and promote cell proliferation (labeled by Ki67) at Day 8 to actively intervene in the wound healing process. Figure 7j shows the immunohistochemical staining images on Day 16, and the quantitative results are shown in Figures S24a–c and S25a,b (Supporting Information). GFM/N could recruit M2 macrophage and promote collagen deposition and collagen remodeling. In particular, the functions of M2 macrophages can be summarized into three aspects: phagocytosis, inflammation regulation, and tissue remodeling.^[^ ^54^ ^]^ They play an indispensable role in the process of skin repair.

A subcutaneous transplantation model of SD rats was also established to evaluate the biocompatibility of GFM microneedle. As shown in Figure S26 (Supporting Information), a dense capsule formed around the materials, owning to the immune rejection reaction. No significant inflammatory cell infiltration and tissue necrosis were found among the three groups, indicating good tissue compatibility. Fresh blood was collected from the treated animals and detected by a series of biochemical assays. As shown in Figure S27a–h (Supporting Information), each index exhibited no significant difference between the four groups (*P >* 0.05), suggesting good hemocompatibility. The organs of treated animals, including the brain, heart, liver, spleen, lung, and kidney, were also resected and detected by H&E staining assay. As shown in Figure S28 (Supporting Information), all groups exhibited good organ compatibility. In conclusion, the biocompatibility of the GFM microneedle itself could meet the requirements of medical devices in vivo.^[^ ^55^, ^56^ ^]^

## 3 Conclusion

This study developed a Fe_3_O_4_/MXene (FM) heterojunction‐laden, double‐layer GelMA microneedle. The results demonstrated that FM heterojunction was first synthesized using a hydrothermal method. Compared to monolayer Ti_3_C_2_ nanosheets, FM heterojunction exhibited better photothermal conversion efficiency and cascading nanozyme‐like activities under neutral and weakly alkaline conditions. The incorporation of FM heterojunction could endow the composite GFM microneedle with enhanced broad‐spectrum antibacterial and anti‐oxidative activities, which are both vital for diabetic wound healing. This study also revealed the mechanisms of bacterial ferroptosis from an antibacterial perspective and its relationship with the vacancy defect of FM heterojunction. The products of this study could not only accelerate *S. aureus*‐infected wound healing in an STZ‐induced diabetic model but also exhibit good biocompatibility in a subcutaneous transplantation model. Notably, their pro‐regenerative efficacy surpassed that of commercial antibacterial dressing from Contavec Co., Ltd. Thus, this study provides an effective and safe microneedle material with the potential for the clinical treatment of diabetic wounds or other wounds.

## 4 Experimental Section

### Materials

Bulk Ti_3_AlC_2_ was purchased from XFNANO Materials Tech Co., Ltd (Nanjing, China). Gelatin, methacrylic anhydride (MA), and 5,5‐dimethyl‐1‐pyrroline N‐oxide (DMPO) were obtained from Macklin Inc. (Shanghai, China). Ferrous chloride (FeCl_2_), ferric chloride (FeCl_3_), LiCl, HF, hydrochloric acid (HCl), ammonia, and photo‐initiator were obtained from Sinopharm Chemical Reagent Co., Ltd. (Shanghai, China). Mouse fibroblasts (L929 cells) and human umbilical vein endothelial cells (HUVEC) were kindly provided by the Medical Research Center, Zhongnan Hospital of Wuhan University. Cell medium, trypsin, fetal bovine serum (FBS), and phosphate‐buffered saline (PBS) were purchased from Thermo‐Fisher Scientific Inc. (Shanghai, China). Luria‐Bertani (LB) bacteria medium, H_2_O_2_, cell apoptosis kit, ROS assay kit, and bacterial live/dead staining kit were purchased from Beyotime Biotechnology (Shanghai, China). LPO assay kits were purchased from Sangon Biotech. Co., Ltd (Shanghai, China). Other chemicals and commercial kits were used as received.

### Preparation of FM Heterojunction—Etching of monolayer MXene

Monolayer Ti_3_C_2_ nanosheets were obtained using an improved etching method. ^[^ ^57^ ^]^Briefly, 1 g of bulk Ti_3_AlC_2_ was added into 100 mL of etching solution containing 50 wt.% HF and 12 mol/L HCl. The mixture was reacted at room temperature for at least 12 h and then centrifuged at 3000 rpm for 60 s. The precipitate was collected and rinsed with distilled water until the pH value reached 7.0. The precipitate was then mixed with 5 wt.% LiCl solution, reacted with constant stirring for another 12 h, and centrifuged to remove the supernatant. The obtained precipitate (coded as Ti_3_C_2_ nanosheets) was rinsed at least 3 times and freeze‐dried for further study.

### Preparation of FM Heterojunction—Synthesis of FM heterojunction

Fe_3_O_4_/MXene heterojunction was synthesized using a hydrothermal reaction. FeCl_2_ and FeCl_3_ powder, with a molar ratio of 1: 1.5, was dissolved into distilled water. A small amount of Ti_3_C_2_ nanosheets was added into the precursor solution, followed by ultrasonic oscillation for 30 min. This reaction took place in a three‐necked flask. In a nitrogen atmosphere, the flask was heated to 80 °C. Ammonia was added to the flask until the pH of the mixture reached 10. Hydrothermal reaction occurred at 80 °C for 3 h. After that, FM heterojunction was obtained by centrifugation and rinsed with distilled water 3 times. Herein, neat Fe_3_O_4_ nanoparticles were prepared using the same protocols and used as the control.

### Fabrication of FM‐Laden Biomaterials—‐Preparation of FM‐laden hydrogel

According to our previous report,^[^ ^38^ ^]^ GelMA was chemically modified from gelatin and used as the photo‐responsive substrate of composite biomaterials. Herein, GelMA solution was obtained by dissolving 1.5 g of GelMA into 10 g of distilled water. After that, 20 mg of FM heterojunction and 100 µL of 10 wt.% photo‐initiator solution were added, followed by constant stirring for 30 min. The mixed solution was centrifuged to remove the air bubbles, poured into molds, and cross‐linked by UV light with a power of 500 W for 5 min. The obtained GelMA/FM heterojunction composite hydrogel was coded as GFM hydrogel. As the controls, neat GelMA hydrogel, GelMA/Ti_3_C_2_ hydrogel, and GelMA/Fe_3_O_4_ hydrogel were also prepared using the same protocols and coded as GM hydrogel, GT hydrogel, GF hydrogel, respectively.

### Fabrication of FM‐Laden Biomaterials—‐Preparation of FM‐laden microneedle

Herein, an FM‐laden GelMA microneedle (coded as GFM) with a double‐layer structure was designed. The base layer was composed of neat GM hydrogel, and the tip layer was composed of GFM hydrogel. The microneedles were prepared using a two‐step casting method. The mold used in this study was customized by Xinyun Nanotechnology (Suzhou) Co., Ltd. The protocols for fabricating the double‐layer microneedles can be found in our previous report.^[^ ^40^ ^]^ As the controls, neat GelMA microneedle (GM), double‐layer GelMA/Ti_3_C_2_ microneedle (GT) and GelMA/Fe_3_O_4_ microneedle (GF) were also prepared.

### Physiochemical Characterizations

The morphology of materials was observed using a scanning electron microscope (AMBER GMH, TESCAN, Czech). An energy dispersive spectrometer (EDS) was performed for element detection. The heterojunction was identified using a transmission electron microscope (JEM‐2100, JEOL, Japan). According to standard protocols, the chemical composition of materials was characterized by an X‐ray diffraction spectrometer (Smart‐Lab SE, Rigaku, Japan), X‐ray photoelectron spectrometer (K‐Alpha, Thermo‐Fisher, USA), UV–vis spectrophotometer (Genesys, Thermo‐Fisher, USA), etc. For evaluations of POD‐like activity, electron paramagnetic resonance (EPR) analysis was performed (ELEXSYS E580, Bruker, Germany). In particular, the DMPO was used as the capture agent of hydroxyl radical. A water contact angle test, compression test, swelling test, and skin puncturing test of the composite materials were also performed. More information about the experimental protocols can be found in our previous reports.^[^ ^39^ ^]^

### Others

Please find the additional experimental methods in the Supporting Information.

## Conflict of Interest

The authors declare no conflict of interest.

## Author Contributions

W.Y. performed investigation, methodology, data curation, conceptualization, and wrote the final manuscript. Z.C. performed investigation and visualization. F.X., J.Z., G.W., W.W., and Z.C. performed investigation. W.H. performed supervision and funding acquisition. Y.C. performed supervision, project administration, and funding acquisition. Z.W. performed, validation, supervision, project administration, funding acquisition, and wrote, reviewed and edited the final manuscript.

## Supporting information



Supporting Information

## Data Availability

The data that support the findings of this study are available on request from the corresponding author. The data are not publicly available due to privacy or ethical restrictions.
