# Supplementary material for: Biomolecular Microneedle Initiates Fe3O4/MXene Heterojunction‐Mediated Nanozyme‐Like Reactions and Bacterial Ferroptosis to Repair Diabetic Wounds
Source: Adv Sci (Weinh). 2025 Jan 23;12(11):2417314. doi: 10.1002/advs.202417314 (PMC11923907; doi:10.1002/advs.202417314)
Supplement: Supplementary file 1 — Supporting Information [file ADVS-12-2417314-s001.docx]

**Supporting information**

**Biomolecular microneedle initiates Fe_3_O_4_/MXene heterojunction-mediated nanozyme-like reactions and bacterial ferroptosis to repair diabetic wounds**

*Wenjie You, Zichao Cai, Feng Xiao, Jiaxin Zhao, Guanyi Wang, Wang Wang, Zesheng Chen, Weikang Hu*, Yun Chen*, and Zijian Wang^[[1]](#footnote-1)^**

W. You, G. Wang, W. Wang, Z. Chen, Z. Wang

Department of Urology

Institute of Urology

Cancer Precision Diagnosis and Treatment and Translational Medicine Hubei Engineering Research Center

Zhongnan Hospital of Wuhan University

Wuhan 430071, China

E-mail: [Zijianwang@whu.edu.cn](mailto:Zijianwang@whu.edu.cn)

W. You, Y. Chen

Department of Biomedical Engineering

Hubei Province Key Laboratory of Allergy and Immune Related Disease

TaiKang Medical School (School of Basic Medical Sciences)

Wuhan University

Wuhan 430071, China

E-mail: [yunchen@whu.edu.cn](mailto:yunchen@whu.edu.cn)

W. You, Z. Cai, F. Xiao, J. Zhao

Orthopedic Hospital

Postdoctoral Innovation Practice Base

The First Affiliated Hospital, Jiangxi Medical College

Nanchang University, Nanchang, 330006, China

W. You, W. Hu

School of Materials Science and Engineering

Stem Cells and Tissue Engineering Manufacture Center

Hubei University, Wuhan 430062, China

E-mail: [huwkang@hubu.edu.cn](mailto:huwkang@hubu.edu.cn)

**Photothermal evaluations**

(1) Photothermal test of FM heterojunction

FM heterojunction was suspended in distilled water to prepare a 0.2 wt% FM solution, which was then irradiated with NIR light with a power of 2.5 W/cm^2^ for 240 s. Distilled water served as the blank control, and Ti_3_C_2_ solution with equal concentration served as the positive control. At regular intervals, thermal imaging was captured using an infrared camera (223s, Fortric, China). Dynamic changes in temperature were recorded.

(2) Photothermal test of GFM microneedle

Dried microneedles, including GM, GT, GF, and GFM, were safely placed onto the specimen stages and immediately irradiated by NIR with a power of 2 W/cm^2^. At regular time intervals, thermal images and dynamic changes in temperature were recorded. The power-dependent photothermal effect of GFM was also evaluated with varied power of 1 W/cm^2^, 1.5 W/cm^2,^ and 2 W/cm^2^, respectively. For a cyclic test, the NIR light was turned on for 3 min, turned off for another 3 min, and repeated for 5 cycles.

**Evaluations of nanozyme-like activities**

(1) Peroxidase activity and catalase activity of Fe_3_O_4_ and FM

Phosphate buffer solutions were used to provide simulated conditions of pH 5.8 and pH 7.4. 100 mM H_2_O_2_ solution and DMPO were mixed in buffer with 2mg/mL Fe_3_O_4_ or FM. Electron paramagnetic resonance analysis was used to test the hydroxyl radical for evaluating peroxidase activity. The absorbance value at 652 nm of 3,3',5,5'-Tetramethylbenzidine (TMB) and the absorbance value at 240 nm of H_2_O_2_ were used to detect peroxidase activity and catalase activity in pH 5.8 and pH 7.4.

(2) Hydrogen peroxide scavenging ability

The materials, including nanomaterials and composite microneedles, were incubated with a 100 mM H_2_O_2_ solution. The decomposition reaction occurred with or without NIR irradiation for 10 min. After that, the solution's UV-visible absorption spectroscopy was detected to measure the concentration of H_2_O_2_. At least three independent samples were used for statistical analysis.

(3) Hydroxyl radical scavenging ability

According to a previous report ^[1]^, the 3,3',5,5'-Tetramethylbenzidine (TMB) assay was used to evaluate POD-like activity. The TMB solution was first mixed with FeCl_2_ solution and then incubated with the materials. For some groups, the samples were additionally irradiated with NIR light with a power of 2.5 W/cm^2^. After 10 min of incubation, UV-visible absorption spectroscopy was detected to measure the hydroxyl radical scavenging rate.

(4) Superoxide anion scavenging ability

A pyrogallol autoxidation method was performed as a reliable superoxide-scavenging assay. The materials were incubated with pyrogallol solution with or without NIR irradiation for 10 min. After that, the UV-visible absorption spectroscopy was detected. The absorbance value at 320 nm was used to measure the superoxide anion scavenging rate.

(5) Free radical scavenging ability

The free radical scavenging ability of the materials was evaluated using a 2,2'-casino-bis (3-ethylbenzthiazoline-6-sulfonic acid (ABTS) method. The materials were incubated with an ABTS solution with or without NIR irradiation for 10 min. After that, the UV-visible absorption spectroscopy was detected. The absorbance value at 412 nm was used for quantitative analysis.

**Anti-oxidative evaluations**

L929 cells were treated with a cell medium containing 50 µM of H_2_O_2_ solution for 4 h to establish the cell model of oxidative stress damage. The cells were then incubated with GFM microneedles with or without NIR irradiation to rescue the oxidative stress. After 24 h of incubation, the level of intracellular ROS and cell apoptosis was detected according to the manufacturer’s commercial kit protocols. The migration ability of these cells was evaluated using a scratching assay. An inverted fluorescence microscope (IX73, Olympus, USA) and a flow cytometer (CytoFlex, Beckman, USA) were used for data acquisition.

**Antibacterial evaluations**

(1) Antibacterial phenotypes

In this study, both *E. coli*, *S. aureus,* and MRSA served as the model organisms for broad-spectrum antibacterial evaluations. The proliferative bacteria were suspended in 0.85% normal saline and then incubated with GM and GFM microneedles for 4 h. For the GFM/N group, the bacteria were additionally treated with NIR irradiation with a temperature of 45 ℃ for 5 min. The blank control (B.C.) group was not treated, and the positive control (Amp. or VA) group was treated with antibiotics (ampicillin or vancomycin). The proliferation and survival ability of these bacteria were evaluated by bacterial proliferation assay and clone formation assay. Detailed experimental protocols can be found in our previous report ^[2]^. The treated bacteria were fixed with 4 wt% polyformaldehyde for 30 min, dehydrated with a gradient concentration of alcohol, and coated with a gold layer. The morphology of bacteria was observed using a scanning electron microscope. A live/dead bacteria staining assay was performed according to the manufacturer’s protocols. At least three independent samples were used for statistical analysis.

(2) Mechanism investigations

Prokaryotic transcriptome sequencing was performed to screen the molecular mechanism of antibacterial activity. *S. aureus* was treated with GFM microneedles combined with NIR irradiation. The control group was not treated. After that, the bacteria were lysed and detected with the help of Novogene Technology Co., Ltd (Beijing, China) as follows:

Total RNA was isolated using the Trizol Reagent, after which the concentration, quality, and integrity were determined using a NanoDrop spectrophotometer. Sequencing libraries were generated using the TruSeq RNA Sample Preparation Kit. Firstly, mRNA was purified from total RNA using poly-T oligo-attached magnetic beads. Fragmentation was carried out under elevated temperatures in an Illumina proprietary fragmentation buffer. First strand cDNA was synthesized using random oligonucleotides and Super Script II. Second strand cDNA synthesis was subsequently performed using DNA Polymerase I and RNase H. Remaining overhangs were converted into blunt ends via exonuclease/polymerase activities and the enzymes were removed. After adenylation of the 3′ ends of the DNA fragments, Illumina PE adapter oligonucleotides were ligated to prepare for hybridization. To select cDNA fragments of the preferred 400-500 bp in length, the library fragments were purified using the AMPure XP system. DNA fragments with ligated adaptor molecules on both ends were selectively enriched using Illumina PCR Primer Cocktail in a 15 cycle PCR reaction. Products were purified and quantified using the Agilent high sensitivity DNA assay on a Bioanalyzer 2100 system. The sequencing library was then sequenced on the NovaSeq Xplus platform.

De nove transcriptome analysis was performed as follows:

1. Quality control

Samples are sequenced on the platform to get image files, which are transformed by the sequencing platform software. The original data in FASTQ format (Raw Data) is generated. Sequencing data contains a number of connectors and low-quality Reads, so we use fast (0.22.0) software to filter the sequencing data to get high-quality sequences (Clean Reads) for further analysis.

2. Transcriptome assembly

For the transcriptome sequencing project without a reference genome, we use Trinity (v2.15.1) software to montage Clean Reads for the transcripts for analysis later. After the montaging is completed, FASTA-format Transcript sequence files can be obtained. The longest transcript of each gene was extracted as the representative sequence of the gene, called Unigene.

3. Gene functional annotation

We have annotated gene function for Unigene. The database used in gene function annotation includes NR (NCBI non-redundant protein sequences), GO (Gene Ontology), KEGG (Kyoto Encyclopedia of Genes and Genome), eggnog (evolutionary genealogy of genes: Non-supervised Orthologous Groups), Swiss-Prot, Pfam.

4. Expression analysis:

RSEM (v2.15) statistics were used to compare the Read Count values on each gene as the original expression of the gene, and FPKM was used to standardize the expression.

5. Differential expression analysis:

Next, we used DESeq (v1.38.3) to analyze the genes of difference expression with screened conditions as follows: expression difference multiple |log2FoldChange| > 1, significant P-value < 0.05. At the same time, the R language Pheatmap (v1.0.12) software package was used to perform a bi-directional clustering analysis of all different genes of samples. We get a heat map according to the expression level of the same gene in different samples and the expression patterns of different genes in the same sample using the Euclidean method to calculate the Complete Linkage method to cluster.

6. GO and KEGG enrichment analysis:

We mapped all the genes to Terms in the Gene Ontology database and calculated the numbers of differentially enriched genes in each Term. Using topGO (v2.50.0) to perform GO enrichment analysis on the differential genes (all DEGs / up DEGs / down DEGs), calculate the P-value by hypergeometric distribution method (the standard of significant enrichment is P-value <0.05), and find the GO term with significantly enriched differential genes to determine the main biological functions performed by differential genes. ClusterProfiler (v4.6.0) software was used to carry out the enrichment analysis of the KEGG pathway of differential genes, focusing on the significant enrichment pathway with a P-value <0.05.

The bacterial ferroptosis state was visualized using an LPO staining kit. The contents of intercellular ferrous ion, malondialdehyde (MDA), and glutathione (GSH) were also detected using commercial kits. An inhibitor of ferroptosis (ferrostatin-1) was used for a series of rescue assays. The releasing dynamics of Fe^2+^/Fe^3+^ ions were detected using an inductively coupled plasma optical emission spectrometer (iCAP™ 7400, Thermo-Fisher, USA). A titration assay of KMnO_4_ was performed to determine the ratio of ferrous iron and ferric iron.

A study on the density functional theory (DFT) of the defect nature of FM heterojunction was performed with the help of Yanqu Information Technology Co., Ltd (Hangzhou, China). All the calculations are performed in the framework of the DFT with the projector-augmented plane-wave method, as implemented in the Vienna ab initio simulation package. The generalized gradient approximation is selected for the exchange-correlation potential. The DFT-D3 approach describes the long-range van der Waals interaction. The cut-off energy for the plane wave is set to 500 eV. The energy criterion is set to 10^-5^ eV in the iterative solution of the Kohn-Sham equation. A vacuum layer of 30 Å is added perpendicular to the sheet to avoid artificial interaction between periodic images. The Brillouin zone integration is performed using a 3x3x1 k-mesh. All the structures are relaxed until the residual forces on the atoms have declined to less than 0.03 eV/Å.

**Wound healing evaluations**

This study was carried out with the approval of the Animal Welfare and Ethics Committee of Hubei University (NO. 20240034). Twenty-four female Sprague-Dawley (SD) rats weighing about 200 g were kindly provided by the Biotechnology Research Center of China Three Gorges University. All rats were conventionally fed in a specific pathogen-free (SPF) environment to minimize any external stress. According to a previous report ^[3]^, a diabetic model of SD rats was successfully established by streptozotocin (STZ) injection.

All animals were randomly divided into 4 groups (n = 6). Before surgery, they were safely anesthetized by isoflurane inhalation, and their back hair was removed. After disinfecting a 75% alcohol solution three times, a square full-thickness skin defect was created on each rat and then inoculated with *S. aureus* suspension*.* The negative control group was not treated, and the positive control group was treated with an Ag-based wound dressing (AQUACELAg Advantage, Contavec, England). The GFM group was treated with GFM microneedles, and the GFM/N group was treated with GFM microneedles and photothermal therapy at the same time.

All animals were conventionally fed for another 16 days to allow skin regeneration. At each time point, the wounds were photographed, and the wound area was measured. At day 8, wound exudate was collected for bacterial culture. At day 8 and day 16, neo-skin tissues were collected for a series of histological analyses, including H&E staining, immunofluorescence staining of ROS, iNOS, HIF-1α, CD45, and Ki67, immunohistochemical staining of CD86, CD206, Col-I, and Col-III. According to standard protocols, histological analysis was performed with the help of Servicebio Biotechnology Co., Ltd (Wuhan, China). The images were captured using a laser confocal microscope (Stellaris 5, Leica, Germany).

**Biocompatibility evaluations**

A subcutaneous transplantation model of SD rats was established for the biocompatibility evaluations *in vitro*. Twelve female SD rats weighing about 200 g were randomly divided into four groups (n = 3). GM, GT, and GFM groups were transplanted with GM hydrogel, GT hydrogel, and GFM hydrogel, respectively. The blank control (B.C.) group was sham-operated. All animals were conventionally fed for another 14 days. After that, fresh whole blood was collected for a series of blood biochemical tests, which were performed with the help of the Clinical Laboratory of Zhongnan Hospital. The hydrogels, surrounding capsule tissues, and the organs (brain, heart, liver, spleen, lung, and kidney) were also collected for histological analysis.

**Statistical analysis**

Quantitative data were measured using SPSS software, and the results are shown as means ± standard deviation. Statistical analysis was performed by one-way ANOVA with at least three independent samples. *P* < 0.05 indicated a significant difference.

**References**

[1] M. Qin, X. M. Zhang, H. Y. Ding, Y. B. Chen, W. X. He, Y. Wei, W. Y. Chen, Y. K. Chan, Y. W. Shi, D. Huang, Y. Deng, *Adv. Mater.* **2024**, 36, 18.

[2] Z. J. Wang, M. F. Ke, L. He, Q. Dong, X. Liang, J. Rao, J. J. Ai, C. Tian, X. W. Han, Y. N. Zhao, *Regen. Biomater.* **2021**, 8, 12.

[3] H. Yang, D. M. Lv, S. Q. Qu, H. L. Xu, S. T. Li, Z. Y. Wang, X. L. Cao, Y. C. Rong, X. H. Li, H. L. Wu, Y. F. Chen, J. Y. Zhu, B. Tang, Z. C. Hu, *Advanced Science* **2024**, 21.


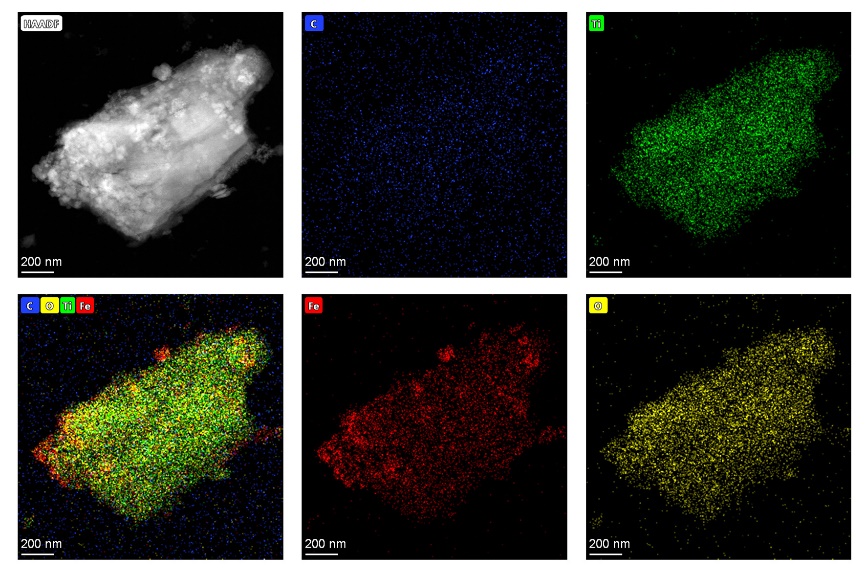


**Fig. S1.** TEM mapping of FM heterojunction. Scale bar: 200 µm.


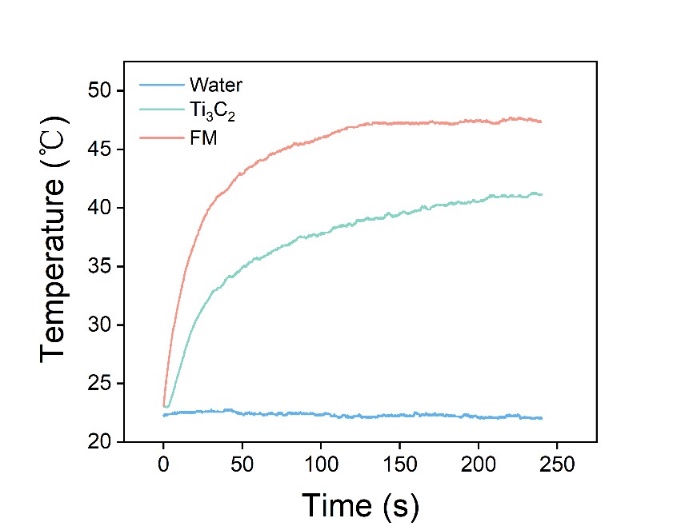


**Fig. S2.** Photothermal curves of Ti_3_C_2_ and FM heterojunction *in vitro*.


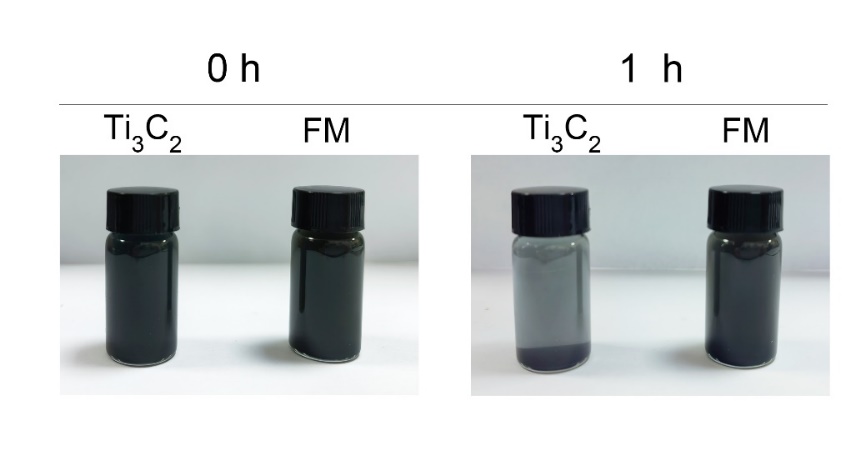


**Fig. S3.** The images of Ti_3_C_2_ solution and FM solution before and after 1 h of settlement.


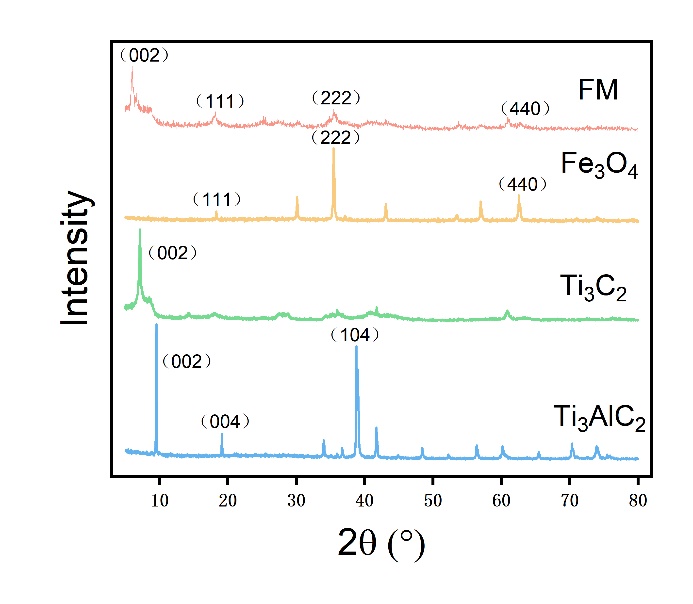


**Fig. S4.** XRD spectrum of FM heterojunction, Fe_3_O_4_ nanoparticles, Ti_3_C_2_ nanosheets and bulk Ti_3_AlC_2_. The characteristic peaks were marked.


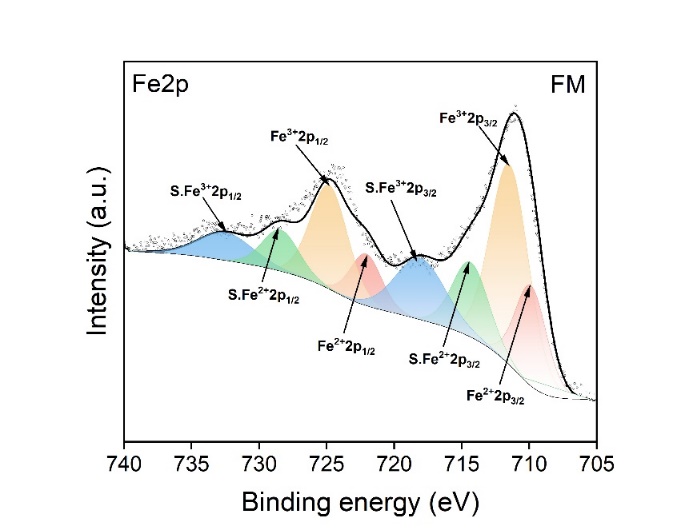


**Fig. S5.** XPS spectrum of FM heterojunction. The characteristic peaks were marked.


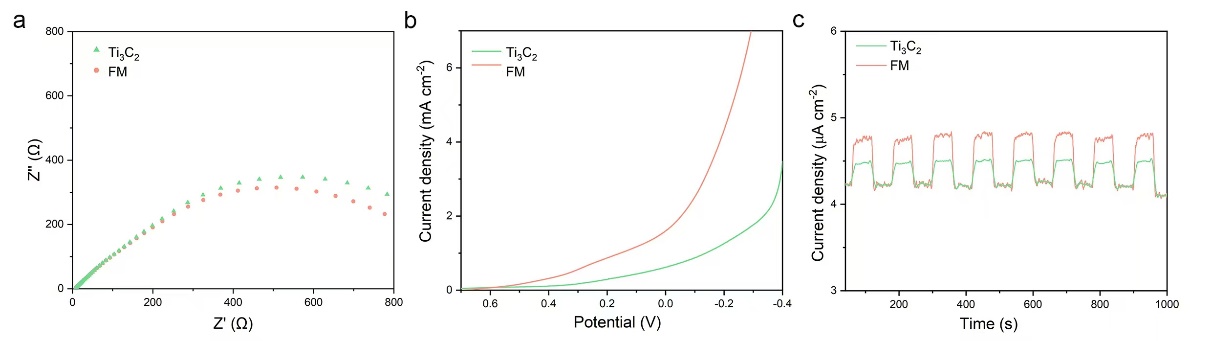


**Fig. S6.** (a) Electrochemical impedance spectroscopy of FM and Ti_3_C_2_; (b) Linear scanning curves; (c) The transient photocurrent curves.


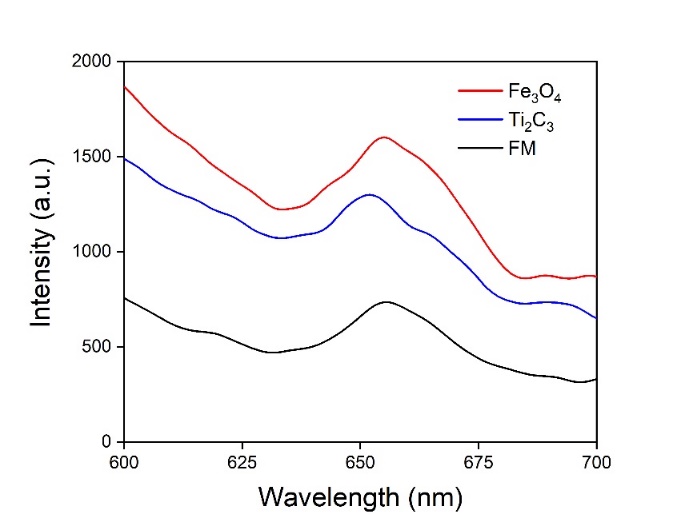


**Fig. S7.** Photoluminescence (PL) emission spectra.


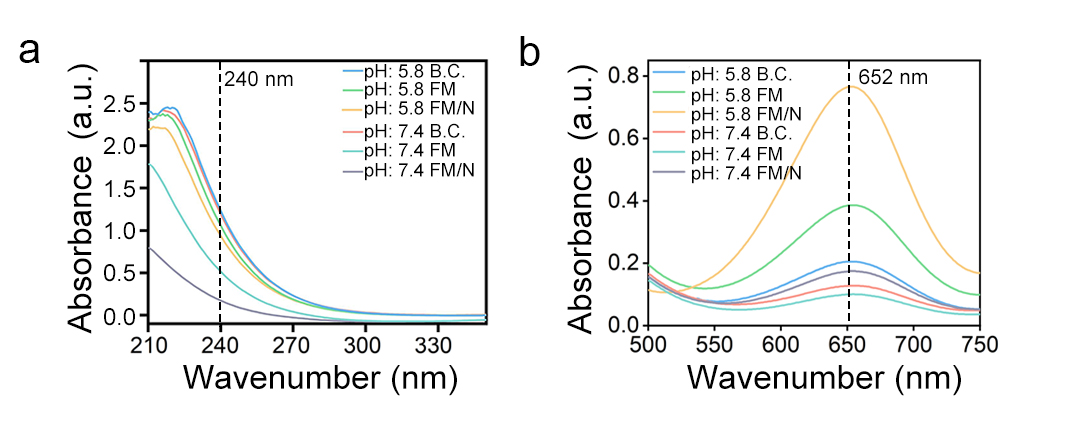


**Fig. S8.** (a) UV-Vis spectrum for the evaluations of CAT-like nanozyme; (b) UV-Vis spectrum for the evaluations of POD-like nanozyme.

**
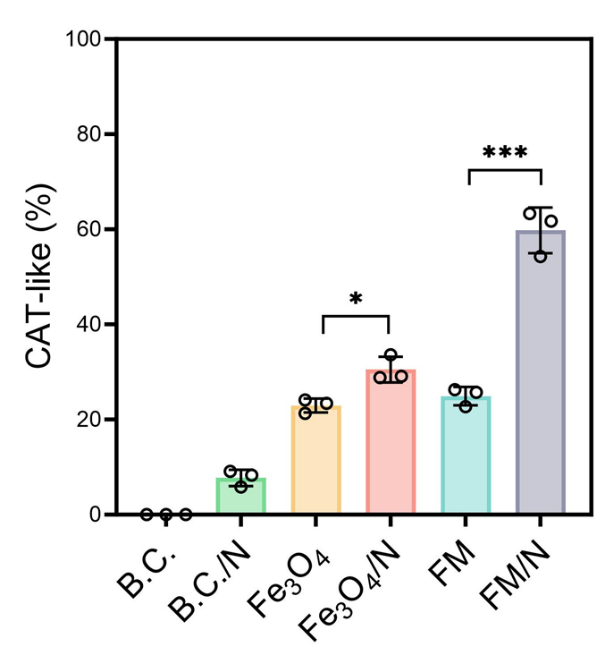
**

**Fig. S9.** The CAT-like activity of FM heterojunction and Fe_3_O_4_ nanoparticles with or without NIR irradiation (*n* = 3). Values are expressed as the mean ± SD. **P* < 0.05, ****P* < 0.001.


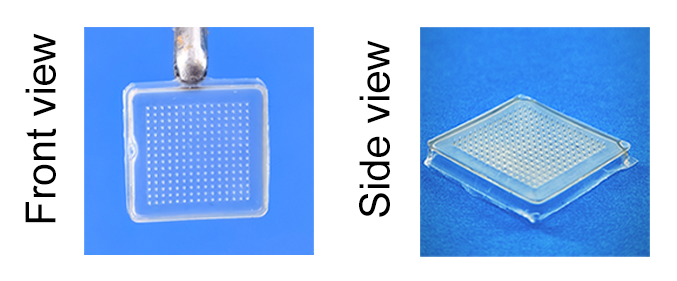


**Fig. S10.** General observation of double-layer GFM microneedles.


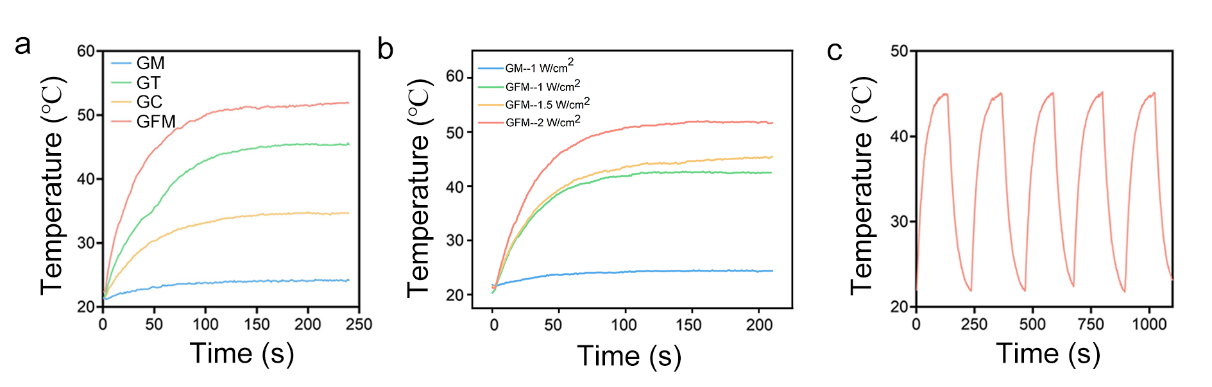


**Fig. S11.** (a) Photothermal curves of varied microneedles; (b) Photothermal curves of GFM microneedle under varied power of NIR light; (c) Cyclic photothermal curve of GFM microneedle.


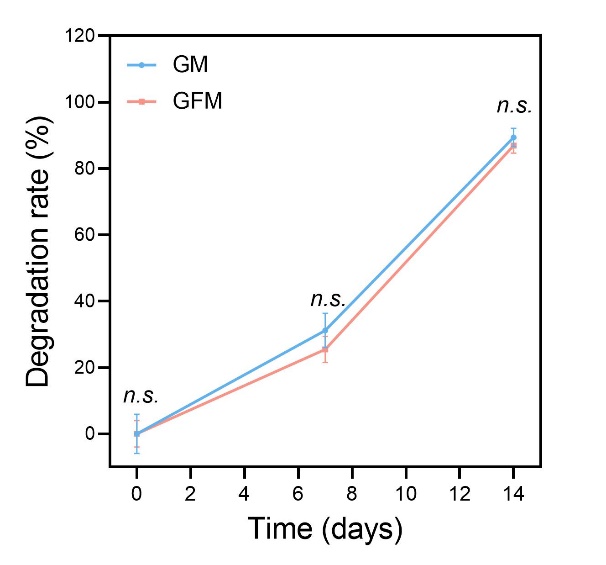


**Fig. S12.** The degradation rate of GM and GFM microneedles *in vitro* (*n* = 3). Values are expressed as the mean ± SD, *n.s.* indicates no significant difference.


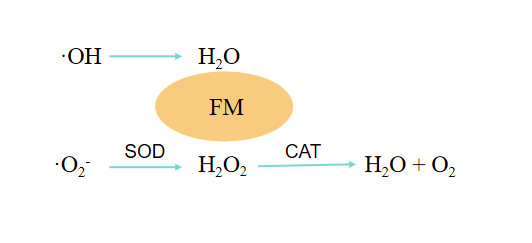


**Fig. S13.** A diagram showing the cascading nanozyme-like activities of FM heterojunction.


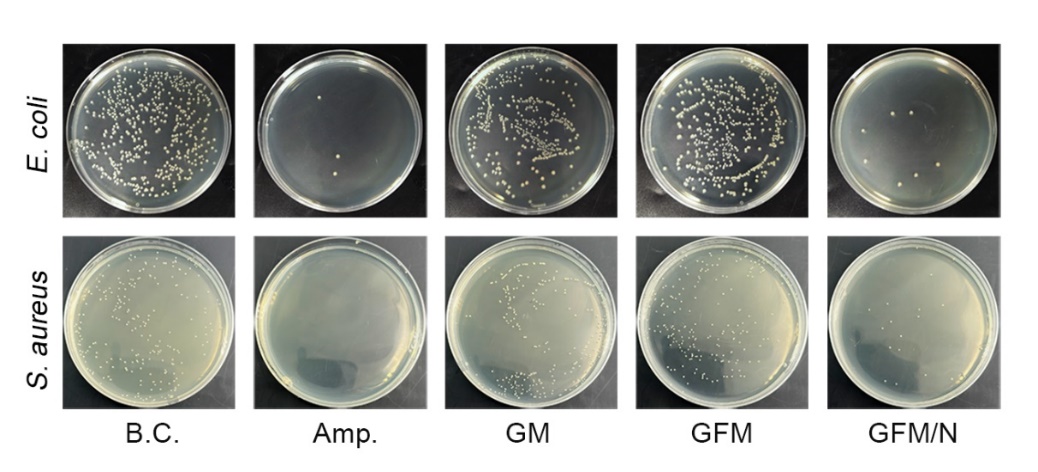


**Fig. S14.** Optical images of bacterial clones for broad-spectrum antibacterial evaluations.


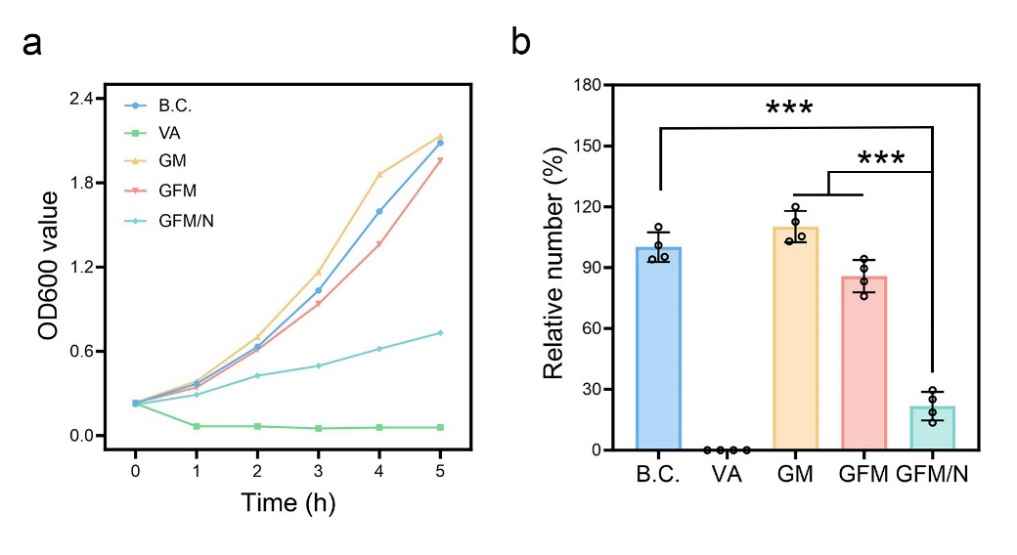


**Fig. S15**. (a) Bacterial proliferation curves of MRSA; (b) Relative number of bacterial clones of MRSA (*n* = 4). Vancomycin (VA) serves as the positive control. Values are expressed as the mean ± SD. ***P* < 0.01, ****P* < 0.001.


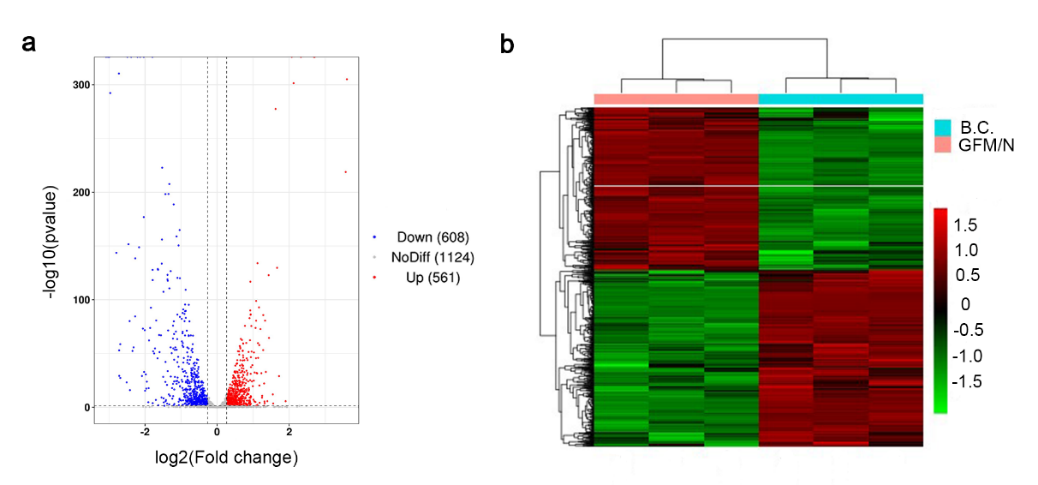


**Fig. S16. (**a) Volcano map of DEGs; (b) Heatmap of DEGs.


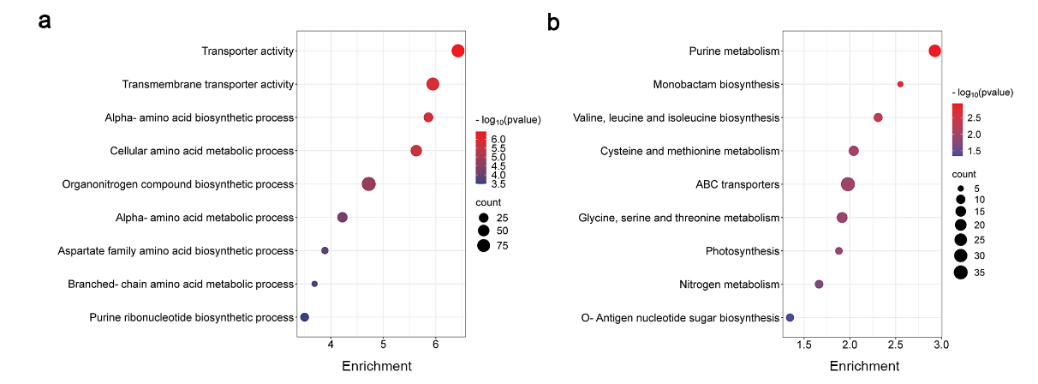


**Fig. S17.** (a) GO analysis of down-regulated DEGs; (b) KEGG analysis of down-regulated DEGs.


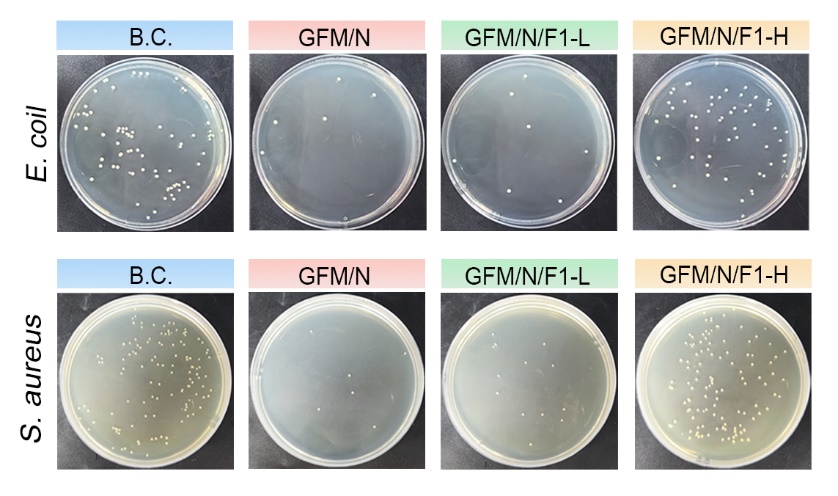


**Fig. S18.** Rescue assay by the inhibitor of ferroptosis (F1). F1-L: low dosage, F1-H: high dosage.


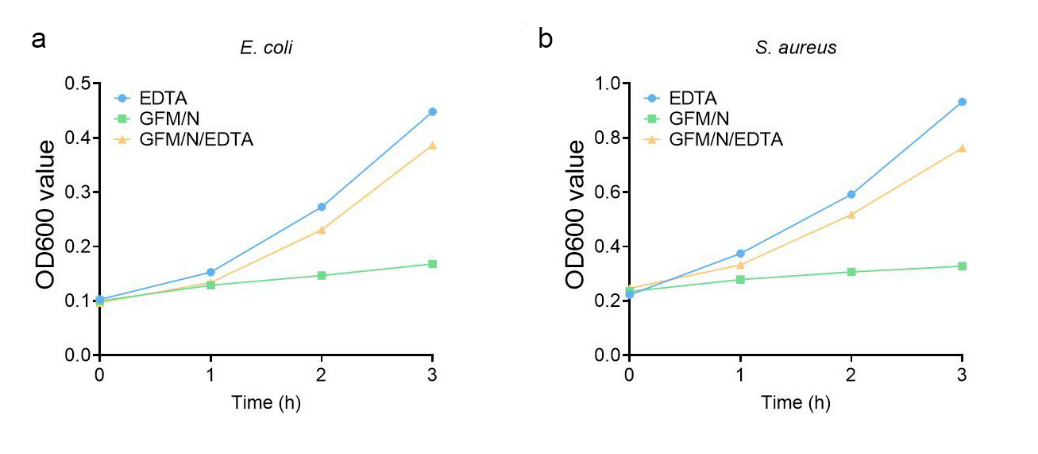


**Fig. S19.** Rescue assay by EDTA. (a) Bacterial proliferation assay of *E. coli*; (b) Bacterial proliferation curves of *S. aureus*.


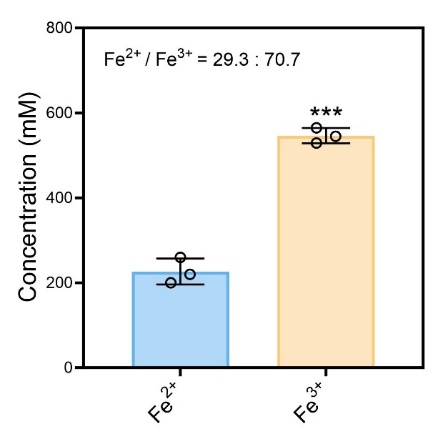


**Fig. S20.** The ratio of ferrous iron and ferric iron released by FM heterojunction (*n* = 3). Values are expressed as the mean ± SD, ****P* < 0.001.


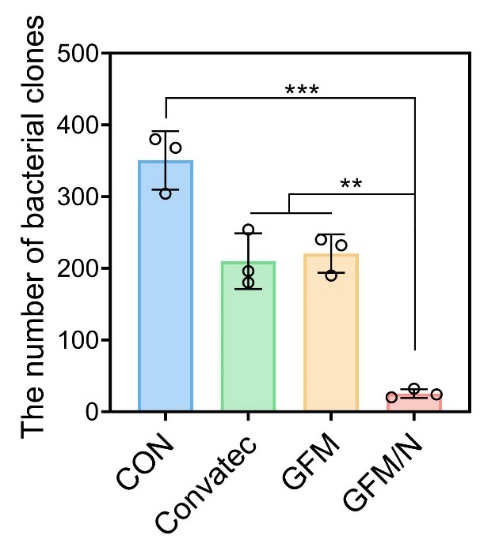


**Fig. S21**. The number of bacterial clones (*n* = 3). Values are expressed as the mean ± SD, Compared to GFM/N group, ***P* < 0.01, ****P* < 0.001.


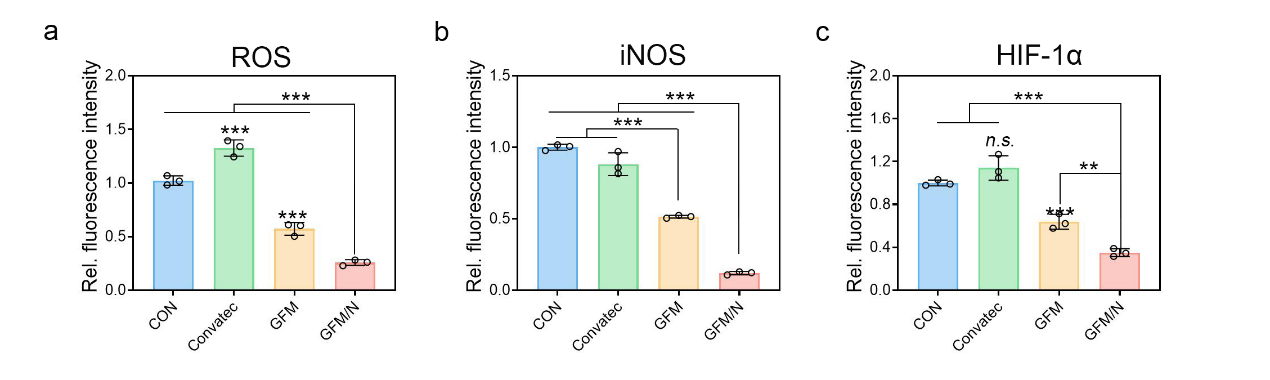


**Fig. S22.** (a) Relative fluorescence intensity of ROS (*n* = 3); (b) Relative fluorescence intensity of iNOS (*n* = 3); (c) Relative fluorescence intensity of HIF-1α (*n* = 3). Values are expressed as the mean ± SD, *n.s.* indicates no significance, ***P* < 0.01, ****P* < 0.001.


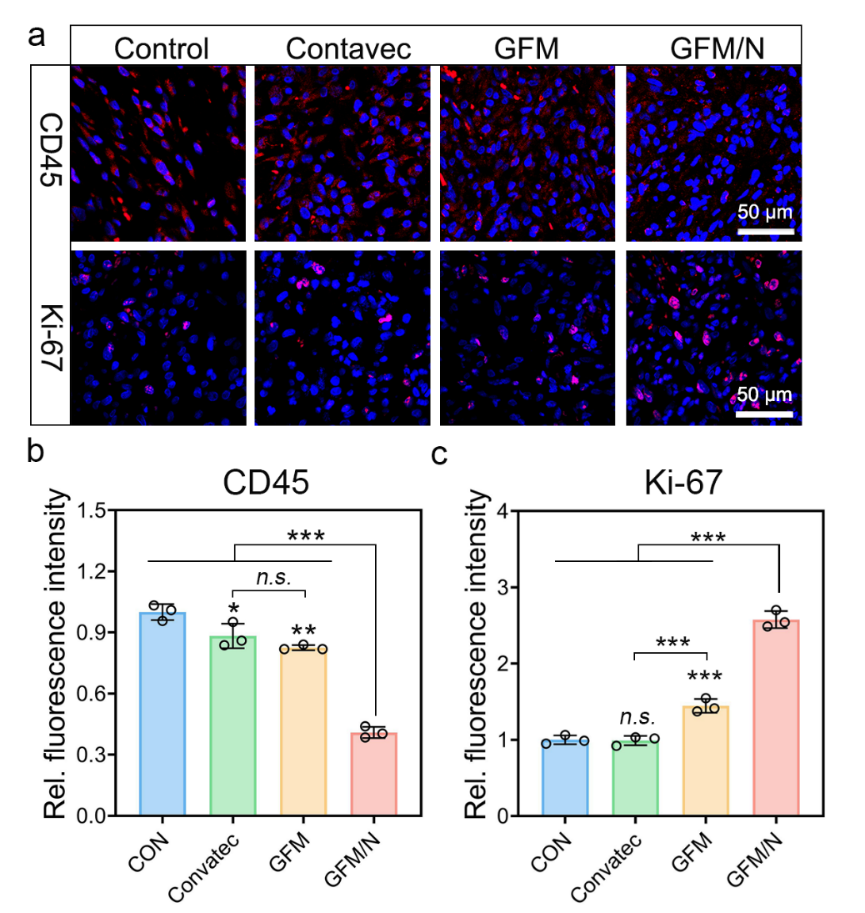


**Fig. S23.** (a) Immunofluorescence staining images of the capsule tissue. Scale bar: 50 µm; (b) Relative fluorescence intensity of CD45 (*n* = 3); (c) Relative fluorescence intensity of Ki-67 (*n* = 3). Values are expressed as the mean ± SD, *n.s.* indicates no significance, ****P* < 0.001.


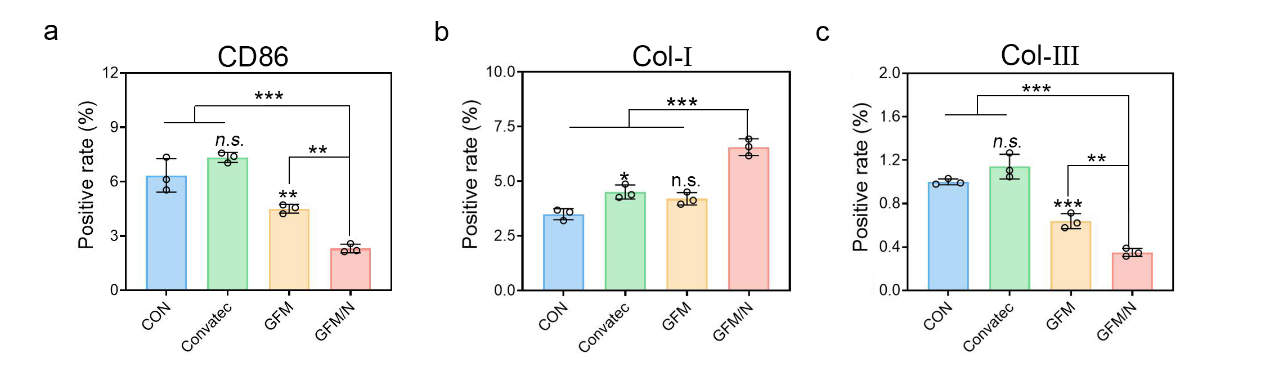


**Fig. S24.** (a) Percentage of CD86^+^ cells (*n* = 3); (b) Percentage of Col-I occupied area (*n* = 3); (c) Percentage of Col-III occupied area (*n* = 3). Values are expressed as the mean ± SD, *n.s.* indicates no significance, ***P* < 0.01, ****P* < 0.001.


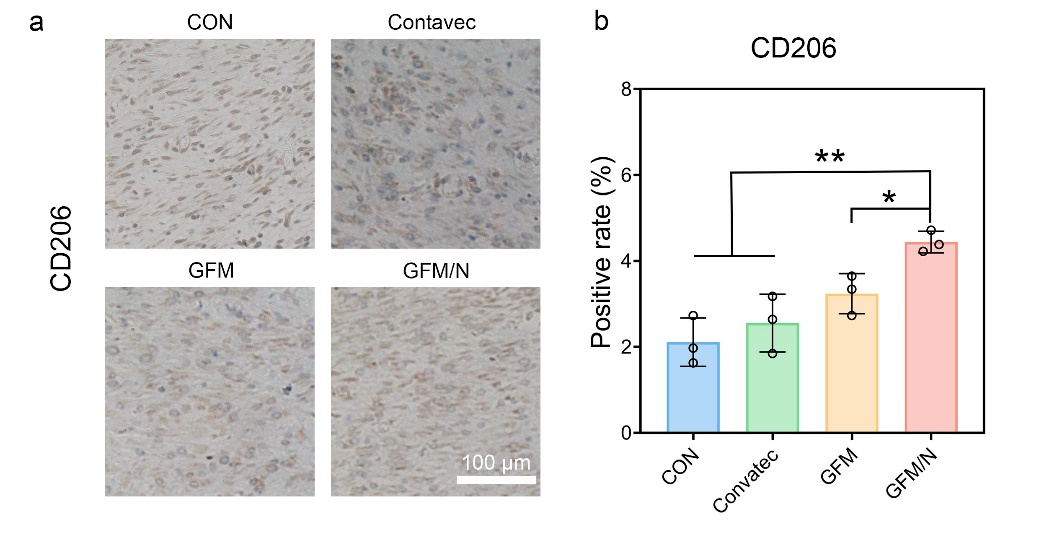


**Fig. S25**. (a) Immunohistochemical staining images of CD206. Scale bar: 100 µm; (b) Quantitative results of CD206 positive rate (*n* = 3). Values are expressed as the mean ± SD, **P* < 0.05, ***P* < 0.01.


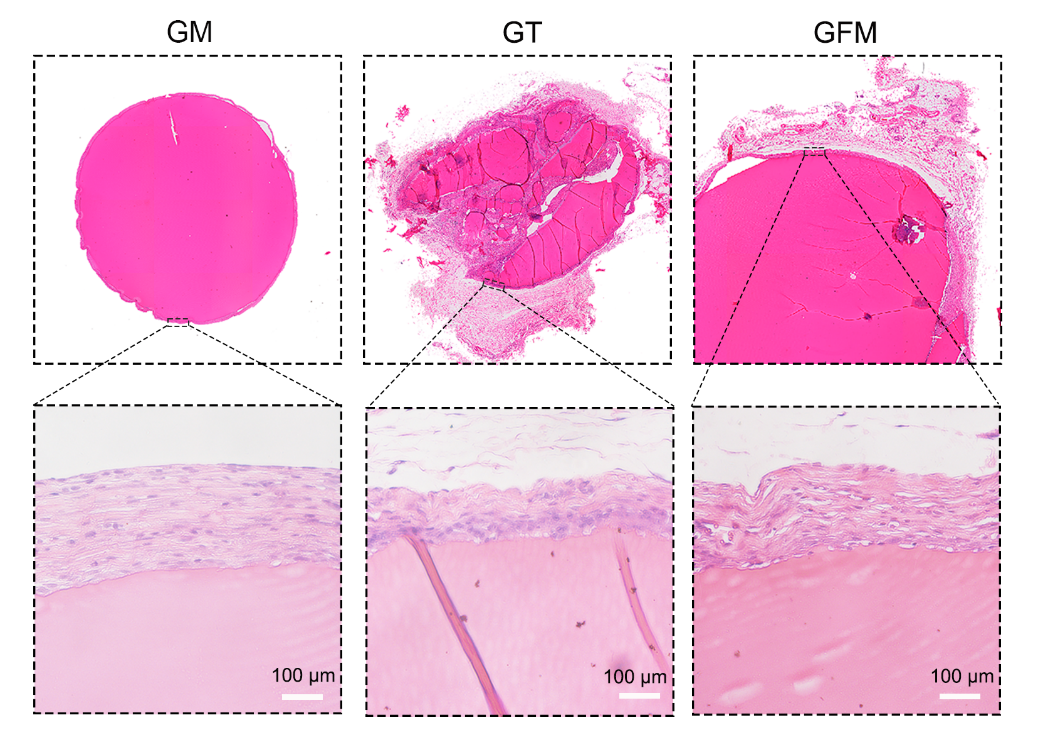


**Fig. S26.** H&E staining images of the materials and surrounding capsule tissue. Scale bar: 100 µm.


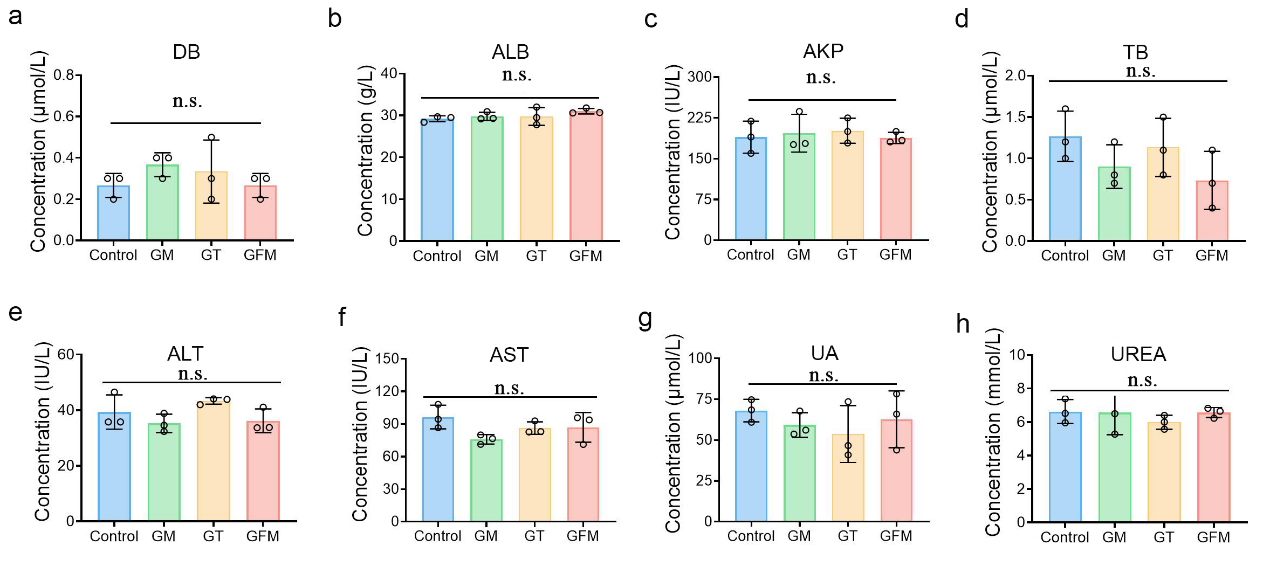


**Fig. S27.** (a-h) Results of a series of blood biochemical indexes (*n* = 3). Values are expressed as the mean ± SD, *n.s.* indicates no significance.


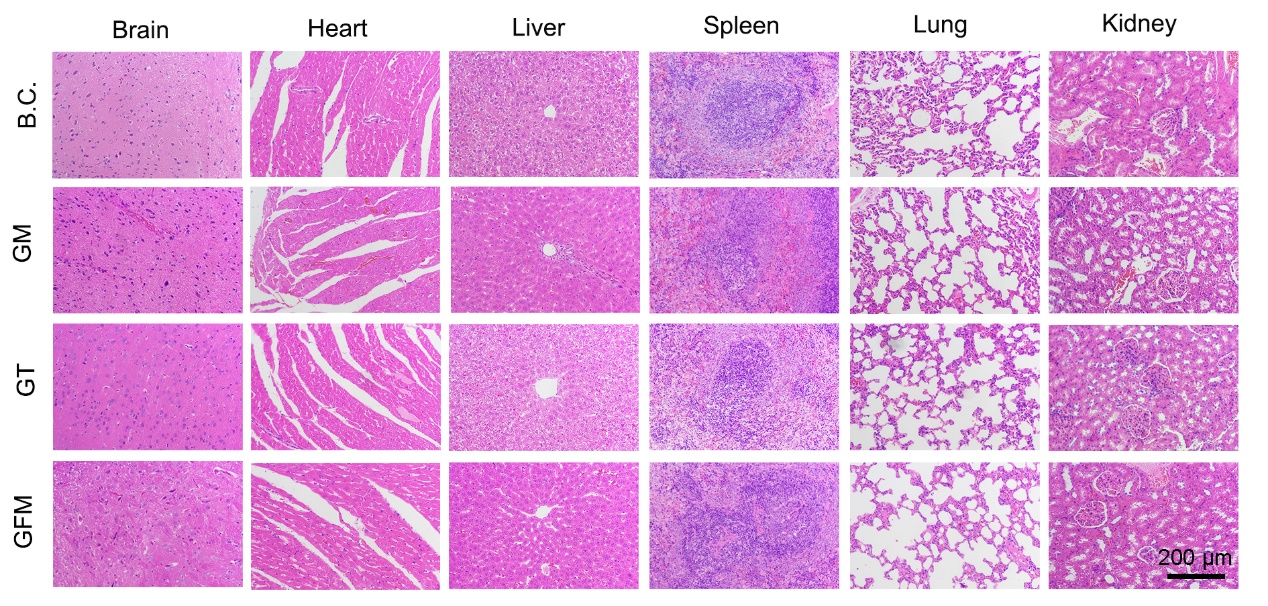


**Fig. S28.** H&E staining images of the organs. Scale bar: 200 µm.

1. [↑](#footnote-ref-1)
